# Risk Factors, Pathological Changes, and Potential Treatment of Diabetes‐Associated Cognitive Dysfunction

**DOI:** 10.1111/1753-0407.70089

**Published:** 2025-04-28

**Authors:** Xiaoyu Meng, Haiyang Du, Danpei Li, Yaming Guo, Peiqiong Luo, Limeng Pan, Ranran Kan, Peng Yu, Yuxi Xiang, Beibei Mao, Yi He, Siyi Wang, Wenjun Li, Yan Yang, Xuefeng Yu

**Affiliations:** ^1^ Division of Endocrinology, Department of Internal Medicine Tongji Hospital, Tongji Medical College, Huazhong University of Science and Technology Wuhan China; ^2^ Hubei Clinical Medical Research Center for Endocrinology and Metabolic Diseases Wuhan China; ^3^ Branch of National Clinical Research Center for Metabolic Diseases Wuhan China; ^4^ Department of Orthopaedics Zhoukou Central Hospital Zhoukou China; ^5^ Department of Endocrinology The Central Hospital of Wuhan, Tongji Medical College, Huazhong University of Science and Technology Wuhan China; ^6^ Computer Center, Tongji Hospital, Tongji Medical College, Huazhong University of Science and Technology Wuhan China

**Keywords:** diabetes, diabetes‐associated cognitive dysfunction, pathological changes, potential drug treatment

## Abstract

**Background:**

Diabetes is a prevalent public health issue worldwide, and the cognitive dysfunction and subsequent dementia caused by it seriously affect the quality of life of patients.

**Methods:**

Recent studies were reviewed to provide a comprehensive summary of the risk factors, pathogenesis, pathological changes and potential drug treatments for diabetes‐related cognitive dysfunction (DACD).

**Results:**

Several risk factors contribute to DACD, including hyperglycemia, hypoglycemia, blood sugar fluctuations, hyperinsulinemia, aging, and others. Among them, modifiable risk factors for DACD include blood glucose control, physical activity, diet, smoking, and hypertension management, while non‐modifiable risk factors include age, genetic predisposition, sex, and duration of diabetes. At the present, the pathogenesis of DACD mainly includes insulin resistance, neuroinflammation, vascular disorders, oxidative stress, and neurotransmitter disorders.

**Conclusions:**

In this review, we provide a comprehensive summary of the risk factors, pathogenesis, pathological changes and potential drug treatments for DACD, providing information from multiple perspectives for its prevention and management.

## Graphical abstract

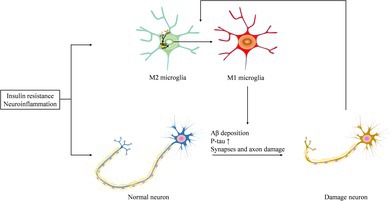

**Summary**

- This study summarizes the methods of measuring cognitive impairment in diabetic patients and analyzes their limitations, providing directions for future research.
- This study provides a detailed analysis of the pathological mechanisms of DACD, emphasizing the roles of insulin resistance, neuroinflammation, microvascular dysfunction, and neurotransmitter disorders in cognitive decline.
- This study evaluates the existing treatment strategies for DACD, such as antidiabetic drugs (GLP‐1 receptor agonists), physical activity, healthy diet (Mediterranean diet), and herbal medicine, and explores potential innovative therapies, offering new perspectives for clinical management.

**Abbreviations**
ACh: Acetylcholine
AChE: Acetylcholinesterase
ACR: Albumin/creatinine ratio
ADDL: Aβ‐derived diffusible ligands
AGE: Advanced glycation end products
APOE: Apolipoprotein E
APP: Amyloid precursor protein
Aβ: Amyloid β
BBB: Blood brain barrier
BDNF: Brain‐derived neurotrophic factor
CNS: Central nervous system
CSF: Serum and cerebrospinal fluid
DACD: Diabetes‐related cognitive dysfunction
DPP‐4i: Dipeptidyl peptidase‐4 inhibitors
DR: Diabetic retinopathy
DTI: Diffusion tensor imaging
eGFR: Estimated glomerular filtration rate
ERK: Extracellular signal‐regulated kinase
Ex‐4: Exendin‐4
GA: Glycoalbumin
GABA: γ‐Aminobutyric acid
GAP‐43: Growth‐associated protein 43
GLP‐1R: Glucagon‐like peptide 1 receptor
GSK3β: Glycogen synthase kinase 3β
HbA1c: Hemoglobin A1c
HFD: High‐fat diet
IR: Insulin resistance
LTP: Long‐term potentiation
MAPK: Mitogen‐activated protein kinase
MCI: Mild cognitive impairment
MDA: Malondialdehyde
MMSE: Mini‐mental state examination
MOCA: Montreal Cognitive Assessment
MRI: Magnetic resonance imaging
NfL: Neurofilament light chain
NF‐κB: Nuclear factor‐κB
NMDA: N‐methyl d‐aspartate
NO: Nitric oxide
PI3K: Phosphatidylinositide 3‐kinase
PPARs: Peroxisome proliferator‐activated receptors
PPARβ/δ: Peroxisomal proliferator‐activated receptor β/δ
PSD 95: Postsynaptic density protein 95
P‐tau: Phosphorylated tau protein
RAGE: Receptor for advanced glycation end products
RCT: Randomized controlled trial
ROS: Reactive oxygen species
SGLT2i: Sodium‐glucose co‐transporter 2 inhibitor
SIRT1: Sirtuin 1
SNAP‐25: Synaptosomal‐associated protein 25
SOD: Superoxide dismutases
STZ: Streptozotocin
SUVR: Standardized uptake value ratio
SYN1: Synapsin‐1
SYP: Synaptophysin
T1DM: Type 1 diabetes mellitus
T2DM: Type 2 diabetes mellitus
TLR4: Toll‐like receptor 4
TREM2: Triggering receptor expressed on myeloid cells 2
T‐tau: Total tau
TZD: Thiazolidinediones

## 1 Introduction

Increasing research indicates that diabetes increases the risk of cognitive dysfunction and eventually dementia in both human and animal models of type 1 diabetes mellitus (T1DM) and type 2 diabetes mellitus (T2DM) [1]. T1DM is an autoimmune disease characterized primarily by decreased insulin secretion, while T2DM is characterized by reduced insulin sensitivity and relative insulin deficiency, accounting for over 90% of all diabetes cases [2]. The chronic hyperglycemia caused by diabetes can lead to both microvascular and macrovascular complications that may impact the brain [2]. Previous studies showed that diabetes raised the risk of cognitive dysfunction by 1.25–1.91 times [3]. The global prevalence of diabetes is expected to rise from 171 million individuals in 2000 to 366 million in 2030 [4]. Meanwhile, the prevalence of dementia is estimated to increase from 24 million in 2001 to 84 million in 2040 [5, 6], suggesting a parallel trend between the increasing burden of diabetes and neurocognitive disorders.

The association between diabetes and cognitive dysfunction is reported in various countries, with some regional variations in prevalence and impact. A large cohort study in the United States reported that individuals with T2DM had a 1.5 to 2‐fold increased risk of developing Alzheimer's disease (AD) and vascular dementia compared to non‐diabetes [7]. A meta‐analysis of observational studies reported that the pooled estimated prevalence of mild cognitive impairment in patients with type 2 diabetes mellitus in China Asia was higher than in Europe [8]. Emerging evidence shows that the impact of diabetes on cognitive function differs between men and women. Two large meta‐analyses including observational and cohort studies reported that the overall prevalence in female patients was higher than that in male patients [8, 9]. The financial burden of caring for dementia patients is 50% higher compared to age‐matched individuals without dementia. Similarly, the cost of managing patients with type 2 diabetes mellitus (T2DM) is 2.5 to 4 times greater than that for non‐diabetic individuals [10]. The cognitive dysfunction caused by diabetes adversely affects the memory and learning abilities of patients, diminishes their quality of life, and heightens the social and national economic burden [11]. However, the pathogenesis and treatments of diabetes‐associated cognitive dysfunction (DACD) remain unclear.

Currently, there is no consensus on the pathophysiological changes or effective treatments of DACD. Cognitive dysfunction further complicates the management of diabetes, making a thorough understanding of DACD essential for developing strategies to prevent or reverse these cognitive complications. In this review, we emphasize the risk factors, pathogenesis, and pathological changes associated with DACD.

For this review, a comprehensive search of the database PubMed was conducted. Search terms included a combination of keywords such as “type 1 diabetes,” “type 2 diabetes,” “diabetes‐associated cognitive dysfunction (DACD),” “cognitive function,” “cognitive dysfunction,” “cognitive decline,” “risk factors,” “pathological mechanisms,” “cognitive impairment,” “dementia,” and “treatment strategy.” Moreover, inclusion criteria included “peer‐reviewed original research articles and reviews,” “studies published in English,” “human and relevant animal studies providing insights into DACD,” “studies with a well‐defined methodology and statistical rigor.” Furthermore, case reports, conference abstracts, and non‐peer‐reviewed studies, articles with insufficient data or unclear methodologies, and studies not specifically addressing cognitive dysfunction in diabetes were excluded.

## 2 Diagnosis of DACD

DACD refers to reduced cognitive performance in diabetic patients, affecting areas like memory, executive function, language, and spatial abilities. However, the performance levels typically do not drop significantly into the abnormal range [12]. Despite this, a definitive standard for evaluating cognitive impairment in diabetic patients has still not been established.

The mini‐mental state examination (MMSE) and the Montreal Cognitive Assessment (MOCA) are two commonly used neuropsychological tests for assessing cognitive function. Higher scores on MMSE and MOCA indicate better cognitive performance, with a threshold of 23/24 on the MMSE for dementia [13] and 25/26 on the MOCA for mild cognitive impairment (MCI) [14]. When assessing cognitive abilities, it is essential to rule out other interfering factors, including speech difficulties, hearing loss, and lack of cooperation. Additionally, metabolic diseases that may temporarily impact cognitive function, like severe hypoglycemia, diabetic ketoacidosis, hyperglycemic hyperosmolar coma, or hypothyroidism, should be considered. Other potential factors include head trauma, conditions affecting brain function, and mental health issues like depression and anxiety, as well as severe lung or kidney diseases, a history of heart failure, and malignancies. A history of substance dependence or abuse should also be noted, particularly if the individual used certain medications in the past month, such as antidepressants, anti‐Parkinson drugs, anti‐epileptic drugs, or sedatives. Lastly, the use of cognitive dysfunction medications like donepezil or memantine must be taken into account [15].

Certain serum and cerebrospinal fluid (CSF) biomarkers are tested in the laboratory to reflect neurodegeneration, including amyloid β (Aβ), total tau (T‐tau), phosphorylated tau protein (P‐tau), as well as presynaptic markers like synaptosomal‐associated protein 25 (SNAP‐25) and growth‐associated protein 43 (GAP‐43), along with axonal markers such as neurofilament light chain (NfL) [16, 17, 18]. However, there is ongoing debate regarding the impact of T2DM on Aβ, T‐tau, and P‐tau levels [19, 20, 21, 22]. Moreover, biomarkers representing vascular integrity, such as angiopoietin and vascular endothelial growth factor, are associated with both T2DM and dementia [23, 24, 25]. Two observational and cohort studies found that plasma levels of vascular growth factor were lower in individuals with T2DM, while reduced CSF concentrations were noted in Alzheimer's disease [26, 27]. However, these proteins often lack specificity for the cerebrovascular bed [28]. Therefore, we recommend that there is a need to identify biomarkers that more accurately reflect the development of cognitive dysfunction in diabetic patients.

Additionally, brain imaging studies help reveal structural damage related to cognitive dysfunction [29, 30]. Moderate brain atrophy, particularly in the hippocampus, is the most frequently documented magnetic resonance imaging (MRI) finding associated with cognitive decline in T2DM [2, 31, 32]. Furthermore, diffusion tensor imaging (DTI) serves as a marker for brain parenchymal injury and assesses global white matter changes [33]. A review reported that DTI studies in T2DM patients showed alterations in white matter microstructure and connectivity compared to controls, which correlated with cognitive dysfunction [34]. However, we consider that it is important to note that fluctuations in osmotic pressure may affect the results of imaging studies in patients with T2DM.

## 3 Risk Factors of DACD (Figure 1)

**FIGURE 1:**
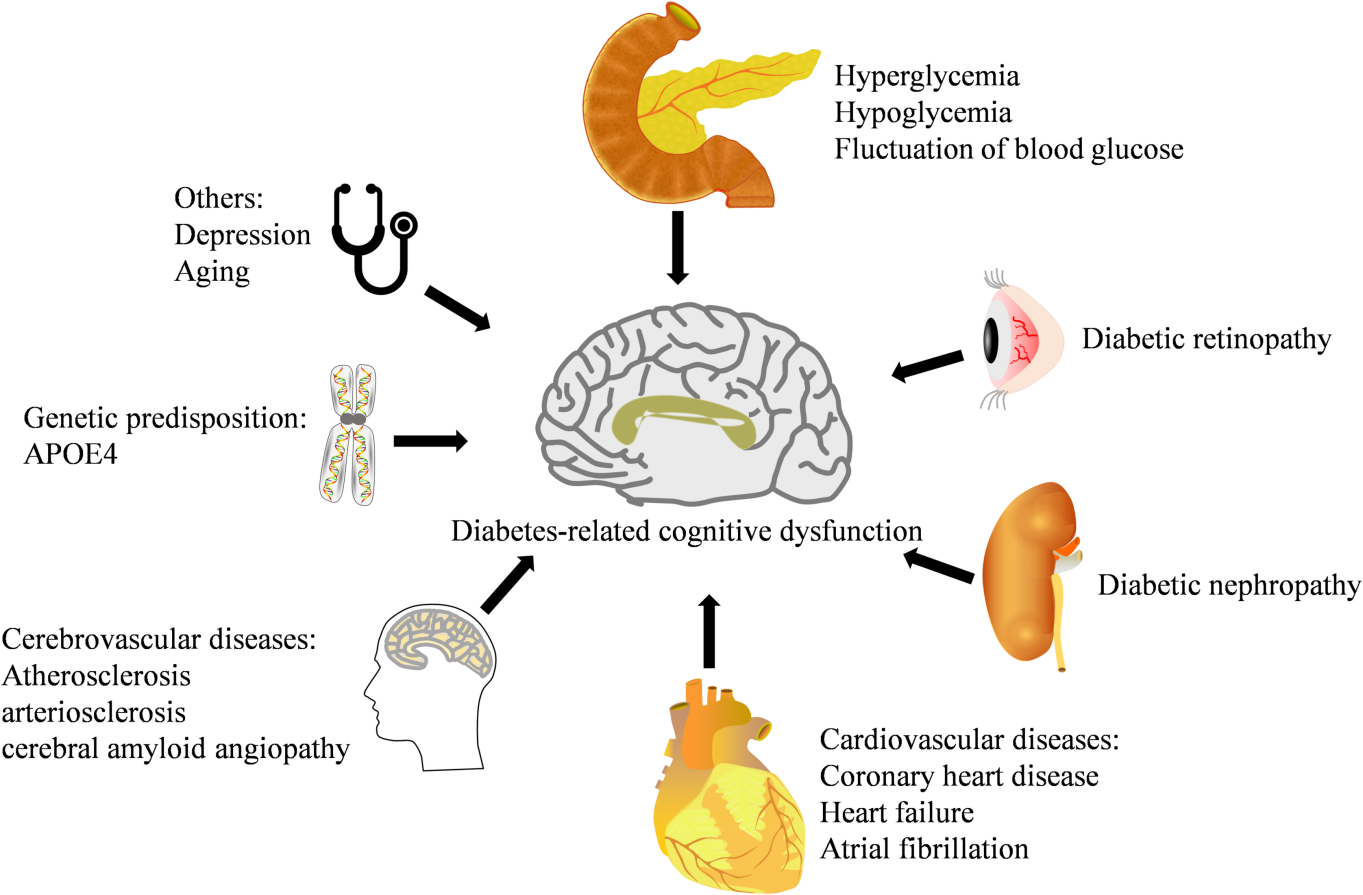
Risk factors of diabetes‐associated cognitive dysfunction. The arrows indicate association.

### 3.1 Hyperglycemia

Hyperglycemia is the most prevalent symptom among diabetic patients. Chronic hyperglycemia contributes to cognitive impairment by inducing oxidative stress, neuroinflammation, and advanced glycation end‐product (AGE) accumulation, leading to neuronal damage [11]. Three clinical studies found that patients with diabetes frequently experience acute and temporary cognitive impairment associated with hyperglycemia, which could impact their quality of life and daily activities [35]. A clinical study reported that the visual reaction time of patients with T1DM was obviously delayed during a clamp study when blood glucose levels reached 16.7 mmol/L [36]. Similarly, blood glucose levels in the 20–30 mmol/L range were associated with a 9.5% decrease in intelligence quotient performance among children with T1DM [37]. Another clinical study conducted a hyperinsulinic glucose clamp study on patients with T2DM at 14.5 and 16 mmol/L, revealing notable disruptions in performance on complex cognitive function tests during hyperglycemic episodes [38]. Additionally, Clinical studies assessed daily cognitive and motor function in patients with T1DM and T2DM, finding that some patients exhibited obvious cognitive dysfunction at a hyperglycemia threshold of approximately 15 mmol/L. While the adverse effects of hyperglycemia varied among diabetic patients, the resulting increase in errors and slower responses during basic verbal and mathematical tasks affected various daily functions, such as managing finances, calculating insulin doses, and performing at school or work [35].

### 3.2 Hypoglycemia

Hypoglycemia, a common occurrence in the stringent management of diabetes, is recognized as an important risk factor for dementia [11]. Recurrent hypoglycemia is associated with impaired neuronal energy metabolism and increased dementia risk due to excitotoxicity and synaptic dysfunction [39]. Two cohort studies established a clear link between episodes of hypoglycemia and cognitive decline in patients with diabetes [40, 41]. Elderly diabetic patients are particularly vulnerable to hypoglycemia due to various comorbidities, undernutrition, polypharmacy, and renal or hepatic damage [42]. In a retrospective study of elderly patients with T2DM, the risk of dementia increased by 26% to 94% with a higher number of severe hypoglycemia episodes [43]. Another observational cohort study found that elderly patients with T2DM who experienced hypoglycemia had a greater risk (HR: 2.689) of dementia compared to those without such episodes [44]. Additionally, diabetic patients with dementia or cognitive dysfunction are much more likely to be hospitalized for hypoglycemia than those with normal cognitive function [45, 46, 47]. However, a prospective cohort and a randomized controlled trial (RCT) found that hypoglycemia does not necessarily heighten the risk of cognitive dysfunction in diabetic patients [48, 49]. This may be attributed to the fact that individuals experiencing more hypoglycemia may be under stricter glycemic control, which may mitigate the neurocognitive damage associated with hypoglycemia [50].

### 3.3 Fluctuation of Blood Glucose

Glucose fluctuations further exacerbate cognitive deficits by triggering vascular endothelial dysfunction and blood–brain barrier disruption [51]. Recent reviews indicated that fluctuations in glucose levels may be linked to cognitive dysfunction as well as dementia risk in diabetic patients [52, 53]. An observational study found that the glycoalbumin (GA) /hemoglobin A1c (HbA1c) ratio, an effective marker for fluctuations of blood glucose levels, was negatively associated with cognitive function scores in elderly patients with T2DM [54]. Similarly, a cross‐sectional, observational study found that varying glucose levels were associated with cognitive impairment in patients with T2DM to some degree [55]. A retrospective study also reported a consensus on this issue [56]. Observational studies found that fluctuations in blood glucose were associated with increased oxidative stress and vascular endothelial dysfunction in T2DM [57], contributing to the onset and progression of dementia [58].

### 3.4 Diabetic Retinopathy

Diabetic retinopathy (DR) is a common microvascular complication of diabetes [59], and an increasing body of research links it to cognitive decline [15, 60]. A longitudinal study found that diabetic patients with DR had over twofold higher odds of experiencing worsening cognitive impairment, while those with moderate or severe DR had threefold higher odds compared to diabetic patients without DR [61]. An observational study identified DR as a contributing risk factor for cognitive dysfunction in T2DM [15]. Moreover, a current meta‐analysis suggested that DR is associated with an increased risk of cognitive dysfunction [62], with a positive correlation between the severity of DR and cognitive dysfunction [63]. Reviews showed a strong connection between retinopathy and cerebral microvascular injury, suggesting that microvascular dysfunction was a key mechanism driving cognitive decline in diabetes [64, 65]. Thus, we hypothesize DR may serve as an important indicator for identifying individuals at high risk of cognitive dysfunction.

### 3.5 Diabetic Nephropathy

Diabetic nephropathy is another common microvascular complication of diabetes, and observational studies found a close relationship between diabetic nephropathy and cognitive function in diabetic patients [66, 67]. A retrospective study found that both the MMSE and MoCA scores were negatively correlated with urinary albumin excretion rates and positively correlated with the estimated glomerular filtration rates (eGFR) in patients with type 2 diabetic nephropathy [68]. Similarly, decreased cognitive function was associated with kidney disease in diabetic patients, as assessed by the albumin/creatinine ratio (ACR), which reflected microvascular endothelial damage, and cystatin C, a marker of eGFR [66]. Studies suggested that cognitive dysfunction related to diabetic nephropathy may arise because brain and kidney damage share similar microvascular lesions [69, 70]. Additionally, the kidneys may serve as the primary clearance pathway for Aβ; the impairment of their function may further aggravate cognitive dysfunction [68, 71].

### 3.6 Cardiovascular Diseases

Systematic reviews consistently showed that patients with a history of cardiovascular diseases, including coronary heart disease, heart failure, and atrial fibrillation, had an increased risk of cognitive impairment [72, 73]. An observational study found that endothelial dysfunction was associated with cognitive impairment in elderly patients with cardiovascular disease [74]. This association is also supported by a prospective study, which found that a history of stroke (OR: 1.523) and cardiovascular diseases (OR: 1.258) was associated with a higher risk of dementia in patients with T2DM [75]. In addition, cardiovascular risk factors, particularly hypertension and dyslipidemia, may contribute to cognitive dysfunction in patients with T2DM [11, 52, 53]. The presence of arterial hypertension in diabetic patients also affects cognitive function [76]. A large network meta‐analysis found that antihypertensive therapy improved cognitive function, except in the language domain [77]. However, lowering blood pressure may potentially reduce cerebral perfusion, leading to an increased risk of cardiovascular complications [78]. Therefore, we think the effectiveness of antihypertensive strategies for preventing cognitive decline remains controversial.

### 3.7 Cerebrovascular Diseases

Cerebrovascular diseases, which often coexist with Alzheimer's disease, include atherosclerosis, arteriosclerosis, and cerebral amyloid angiopathy. Both arteriosclerosis and cerebral amyloid angiopathy fall under the category of small vessel disease, an important vascular contributor to dementia [79, 80]. Chronic hyperglycemia, insulin resistance, and vascular dysfunction contribute to the acceleration of cerebrovascular pathology, increasing susceptibility to cognitive impairment [64]. A RCT showed that in patients with type 1 diabetes and proliferative retinopathy, baseline white matter lesions and reduced skin capillary perfusion were associated with a decline in general cognitive ability over time, regardless of age, sex, HbA1c levels, and severe hypoglycemic events [81]. Moreover, atherosclerosis could lead to cognitive impairment and dementia through thromboembolic stroke in patients with T2DM [28]. An observational study found that diabetes was associated with poor performance on cognitive tests measuring information‐processing speed and executive function, with this relationship partially mediated by markers of cerebrovascular disease [82]. In a cohort study of seniors with diabetes, the risk of dementia was highest (HR: 2.03) among those with a history of cerebrovascular disease [40]. Generally, cognitive decline associated with cerebrovascular disease tends to occur gradually and in a stepwise manner, slowly impacting processing speed, complex attention, and frontal executive function [83].

### 3.8 Genetic Predisposition

Genetics is a vital factor influencing cognitive impairment and Alzheimer's disease [84, 85, 86]. Notably, the 4 allele of apolipoprotein E (*APOE*) stands out as the most reliable and critical genetic risk factor [87]. The effects of *APOE4* include damaged integrity of blood–brain barrier [88], increased accumulation of Aβ [89], and alterations in Aβ metabolism [90]. Moreover, *APOE4* makes the cerebrovascular system more vulnerable to damage from diabetes and exacerbates its effects [91]. A cross‐sectional study found that the impact of diabetes on cognitive performance was more pronounced in individuals carrying one or more APOE ε4 alleles [92]. In a study based on mice, *APOE4* mice exhibited greater vulnerability to the detrimental cognitive effects of high‐fat diet‐induced insulin resistance, likely due to *APOE* subtype‐specific differences in brain metabolism [93]. Furthermore, a clinical cohort study found that rs391300 single‐nucleotide polymorphism in the serine racemase gene, which linked to a higher risk of type 2 diabetes, was also associated with the progression from MCI to probable Alzheimer's disease [94]. However, a population‐based cohort study found that APOE4 genotypes were not associated with cognitive dysfunction in patients with T1DM [95]. More diabetes‐specific genetic studies are needed to clarify these associations and their clinical implications for DACD.

### 3.9 Depression

Patients with diabetes are at an increased risk of developing depression, which is a risk factor for dementia [96, 97]. A systematic review reported that the prevalence of depression among diabetic patients was more than 2–3 times higher than that of those without diabetes [96]. Moreover, a population‐based cohort study showed that the risk ratio for developing dementia in patients with depression was 1.83, for those with diabetes was 1.2, and for those with both conditions was 2.17, indicating that the combined effect of depression and diabetes on dementia risk was greater than simply additive [97]. Another cohort study similarly found that diabetic patients with depression had a two‐fold increased risk of developing dementia compared to those with diabetes alone [98]. Although some debate exists, most structural imaging studies demonstrated that a smaller hippocampal volume was associated with memory dysfunction in depression [99, 100]. However, we think that the underlying cerebral pathology contributing to cognitive dysfunction in depression still requires further investigation.

### 3.10 Other Risk Factors

Beyond the previously mentioned risk factors, several common factors also influence cognitive function in diabetes. Advanced age is a well‐established risk factor for cognitive decline. Two cross‐sectional studies indicated that in patients with T2DM, MMSE score was significantly negatively correlated with age. MMSE score was positively correlated with education level [60, 101]. Education may serve as a protective factor by promoting cognitive reserve, which helps delay the onset of cognitive impairment. Additionally, cognitive decline is positively associated with a longer duration of diabetes [60, 102, 103]. Furthermore, physical inactivity, smoking, and frequent alcohol consumption have also been associated with cognitive impairment in diabetic patients [104, 105]. Sedentary behavior and lack of exercise are also positively associated with cognitive impairment in diabetic patients. Physical activity can improve insulin sensitivity, reduce neuroinflammation, and enhance cognitive function, suggesting that an active lifestyle may mitigate the impact of diabetes on cognition [106]. Moreover, smoking and excessive alcohol consumption accelerate vascular damage and oxidative stress, both of which contribute to DACD [107]. Furthermore, sleep disturbances are also positively associated with impaired cognitive function in patients with T2DM, possibly through mechanisms involving insulin resistance and amyloid deposition [108]. Consequently, these modifiable lifestyle and demographic factors play an important role in the development of DACD. We believe that encouraging healthy behaviors, such as regular physical activity, smoking cessation, moderated alcohol intake, and cognitive engagement, may help reduce cognitive decline in diabetic patients.

## 4 Pathogenesis of DACD

### 4.1 Insulin Resistance

It is widely recognized that insulin mainly functions to regulate blood glucose and energy metabolism in the periphery. Two studies based on rodents found that insulin in the brain promoted the growth and development of nerve cells, regulated the release of neurotransmitters, and played a crucial role in cognitive functions such as learning and memory [109, 110]. Insulin and its receptors are extensively expressed in neurons and glial cells, particularly in the cerebral cortex and hippocampus, areas closely related to cognition [111, 112].

Insulin resistance (IR) is characterized by a decreased sensitivity of insulin in target organs. Prolonged hyperinsulinemia resulting from IR can impair the function of blood–brain barrier (BBB) and insulin activity [113], thereby promoting IR in the brain [112, 114]. Additionally, mitochondrial dysfunction and elevated production of reactive oxygen species, observed in the hippocampus in rodent models of T2DM, also contribute to hippocampal IR. Neuroinflammation is another potential mechanism underlying hippocampal IR in T2DM [30].

Brain IR exposes neurons to high insulin levels for extended periods, resulting in neuronal degeneration and memory impairment [115]. Increasing evidence supports the concept that brain IR is crucial to the pathophysiologic mechanisms of cognitive dysfunction in diabetic patients and may serve as a bridge between diabetes and Alzheimer's disease. A cross‐sectional study found that type 2 diabetic patients developed abnormal functional connectivity in the posterior cingulate cortex, which was associated with insulin resistance in specific brain regions and could play a key role in evaluating the cognitive dysfunction in T2DM [116]. Brain insulin resistance also worsens cognitive function by disrupting brain networks [117]. Notable IR was detected in the hippocampus of diabetic rat models with learning and memory deficits, generated by injection of streptozotocin, and insulin treatment improved the impaired cognitive function in these diabetic rats while increasing the expression levels of phosphorylated insulin receptor and insulin receptor substrate 1 in the hippocampus [118]. Moreover, insulin resistance in hippocampal microvascular may play a critical role in the development of cognitive impairment in T2DM [119]. A study based on mice found that the combination of insulin resistance and hyperinsulinemia did not accelerate plaque formation or memory abnormalities in Alzheimer's disease mice carrying the insulin receptor mutation, but these mutations reduced oxidative damage in mice brains [120]. However, another study based on mice found that mice with a brain‐specific knockout of insulin exhibited brain mitochondrial dysfunction characterized by decreased mitochondrial oxidative activity, increased levels of reactive oxygen species, elevated lipid, and protein oxidation levels in the striatum and nucleus accumbens, along with age‐related anxiety and depressive‐like behaviors [121]. Moreover, brain IR drives pro‐apoptosis, pro‐inflammatory, pro‐phosphorylation of tau protein, and pro‐Aβ cascades [122, 123].

Therefore, the insulin‐signaling pathway is critical for cognitive functions. It primarily activates two key pathways: the phosphatidylinositide 3‐kinase (PI3K)/Akt pathway and the mitogen‐activated protein kinase (MAPK)/extracellular signal‐regulated kinase (ERK) kinase pathway [30, 124]. In high‐fat diet‐induced obesity model mice, exercise improved cognitive function by enhancing the activity of hippocampal insulin signaling (PI3K/Akt) [125]. The activation of the PI3K/Akt pathway phosphorylates and inactivates glycogen synthase kinase 3β (GSK3β), thereby decreasing the ability of GSK3β to phosphorylate the microtubule‐associated protein tau [30]. When insulin signaling is diminished, normal soluble tau levels decrease, while hyperphosphorylated tau accumulates, exacerbating neuronal cytoskeletal collapse, neurite retraction, and impaired synapse formation [126, 127].

We conclude that these suggest that restoring insulin activity in the brain could be an effective strategy to alleviate the cognitive decline associated with T2DM.

### 4.2 Neuroinflammation

Chronic low‐grade neuroinflammation is widely considered a mechanism underlying brain changes in T2DM. Studies based on diabetic rodents found that impaired memory was associated with increased levels of proinflammatory cytokines (TNF‐α, IL‐1β, and IL‐6), while inhibiting neuroinflammation improved cognitive outcomes, suggesting a relationship between neuroinflammation and memory impairment [128, 129, 130, 131]. Causes of nervous system inflammation in diabetes include the entry of peripheral inflammatory cytokines into the brain through disrupted BBB and the activation of microglia triggered by neuronal damage [102, 132, 133]. Several signaling molecules, such as toll‐like receptor 4 (TLR4)/nuclear factor‐κB (NF‐κB) and triggering receptor expressed on myeloid cells 2 (TREM2), play a critical role in regulating microglial activation and neuroinflammation [134, 135, 136]. NF‐κB, a transcription factor involved in the expression of inflammatory cytokines and chemokines, regulated the inflammatory cascade enhancers in brain cells of a mice model of Alzheimer's disease [137]. Moreover, overexpression of TREM2 in the hippocampus reduced neuroinflammation and microglial activation, improving cognitive impairment caused by a high‐fat diet through the NF‐κB signaling pathway [138]. Deficiency of peroxisomal proliferator‐activated receptor β/δ (PPARβ/δ) also involved in the activation of astrocytes and microglia, increasing neuroinflammatory markers [139]. Additionally, lipin 2 upregulation in the hippocampus of diabetic encephalopathy mice reduced NLRP3 inflammasome‐mediated inflammation and improved cognitive function by inhibiting the JNK/ERK signaling pathway [140]. Astrocytes, alongside microglia, are important immune regulatory cells in the central nervous system (CNS), capable of initiating inflammation by secreting cytokines when stimulated [141]. DPP4 (CD26) bound to the insulin‐like growth factor 2‐receptor on Treg cells, impairing Tregs function, polarizing microglia toward a pro‐inflammatory phenotype in the hippocampus, and ultimately leading to neuroinflammation and cognitive impairment in T2DM [142]. Glycation end products induced by hyperglycemia are also implicated in diabetes‐related neuroinflammation. Neuroinflammation can disrupt brain mitochondria and neurotransmitter functions, ultimately leading to neuronal damage and subsequent cognitive decline [143, 144].

### 4.3 Microvascular Dysfunction

The primary role of cerebral microcirculation is to supply nutrients and energy while clearing waste products in response to local neural activity. Cerebral microvascular dysfunction is a common issue in T2DM, and growing evidence suggests it may be a key mechanism in DACD [60, 64]. Two observational studies found that higher scores of microvascular dysfunction—based on MRI‐detected cerebral small vessel disease, retinal arteriolar and venular dilation response to flicker light, albuminuria, and plasma biomarkers—were associated with lower cognitive function [145, 146]. In T2DM, cerebral microvascular pathology is characterized by basement membrane thickening, increased angiogenesis, increased BBB permeability, and altered blood flow regulation, all of which may contribute to cognitive dysfunction [64, 132, 147]. Additionally, cerebral blood flow reduction in T2DM is likely associated with a higher prevalence of cerebrovascular risk factors [148, 149]. Microvascular complications are also associated with cognitive dysfunction in T1DM, where microvascular issues tend to experience both cognitive decline and its acceleration [2, 150]. A review highlighted cerebral microvascular senescence as an important factor in DACD [151].

BBB dysfunction is a significant indicator of cerebral vascular disease. The endothelial function of BBB is essential for regulating ion balance, nutrient transport, blocking neurotoxic molecules, and maintaining brain homeostasis, making it pivotal to the development and progression of DACD [129, 152]. Increased BBB permeability was associated with hyperglycemia and memory deficits in both T1DM and T2DM mouse models [129].

Key drivers of diabetes‐associated cerebral microvascular dysfunction include hyperglycemia, obesity, insulin resistance, and hypertension [64]. Due to similar embryologic and anatomic properties, examining retinal vessels serves as a valuable alternative marker for assessing cerebral microvascular disease [153].

### 4.4 Oxidative Stress

Oxidative stress is recognized as a vital factor in the development and progression of cognitive impairment in diabetes [154]. It impacts insulin resistance, neuroinflammation, neurotransmitters, and lipid metabolism in the diabetic brain [131, 154, 155]. An observational study found that elevated glucose and HbA1c levels were negatively associated with glutathione (GSH) levels in the anterior cingulate of prediabetic/diabetic patients with cognitive impairment, indicating a potential role for oxidative stress in its pathophysiology [156]. In diabetic rats, oxidative and nitrosative stress worked together to damage brain mitochondria through increased reactive oxygen species (ROS) and nitric oxide (NO), and decreased GSH levels [157]. The generation of ROS in the diabetic brain activated multiple cellular pathways, including the AGE, polyol, and protein kinase C pathways, leading to neuroinflammation and neurodegeneration [1, 158]. Superoxide stimulates the production of other ROS and the chelation of NO, disrupting cerebrovascular tension and NO‐dependent dilation. Antioxidant treatments were shown to fully reverse Aβ‐induced cerebrovascular dysfunction in elderly amyloid precursor protein (APP) mice, suggesting oxidative stress as a deleterious mechanism through which Aβ affected cerebral vascular reactivity [159]. Studies suggested that antioxidative stress, including activating sirtuin 1 (SIRT1) [160, 161], Nrf2 [161], GPR55 [162], ChemR23 signaling [163], or carnosine [164], improved cognitive function in diabetic rodent models by reducing inflammatory cytokines and malondialdehyde (MDA) levels while increasing levels of GSH, superoxide dismutase (SOD), and catalase.

### 4.5 Neurotransmitter Disorders

Neurotransmitters are biologically active molecules essential for maintaining the physiological function of the brain, and changes in their metabolism are closely associated with various neurodegenerative diseases [165]. In the CNS, insulin plays a key role in learning and memory by regulating the secretion and reuptake of neurotransmitters, such as acetylcholine (ACh), norepinephrine, and epinephrine, and promoting the accumulation of γ‐Aminobutyric acid (GABA) receptors to the post‐synaptic membrane [1]. Reduced sensitivity to insulin in the hippocampus and cortex can disrupt neurotransmitter synthesis, contributing to cognitive dysfunction. Moreover, pro‐inflammatory mediators may also influence neurotransmitter synthesis [158]. Therefore, we think that alterations in neurotransmitter levels may be central to DACD.

GABA, the primary inhibitory neurotransmitter in the adult mammalian brain, is decreased in DACD [166, 167]. In addition, hyperglycemia made GABAergic neurons in the hippocampus more vulnerable, and their dysfunction, combined with overexcitation of glutamatergic neurons, caused glucose‐induced neurotoxicity during diabetic encephalopathy in mouse models of diabetes [168]. In diabetic rat models, ACh levels were reduced in the hippocampus and hydrolyzed by acetylcholinesterase (AChE) [167]. A study found a significant decrease in ACh in the CSF of diabetic rats with major cognitive damage, which was possibly associated with changes in Aβ42, P‐tau, and IL‐6 [169]. Moreover, increased cholinesterase activities impaired neuronal function, a phenomenon observed in chronic diabetes patients and rodent models, while decreased activities of cholinesterase improved cognitive impairment in diabetic rodent models [170, 171, 172].

Dopamine, a key neurotransmitter, binds to regulatory presynaptic autoreceptors or postsynaptic receptors in the synaptic cleft to trigger action potential, and the presence of diabetes impacts dopaminergic neurotransmission [173]. The increased dopamine clearance and decreased dopamine signaling were observed in rodent models with a brain‐specific knockout of the insulin receptor, suggesting that insulin resistance in the brain altered dopamine turnover [121]. Additionally, obviously reduced levels of serotonin in the hippocampus also contributed to DACD in diabetic rodent models [165, 173, 174]. Furthermore, excitatory neurotransmitters like glutamate and glycine were elevated in DACD [167].

## 5 Pathological Changes of DACD (Figure 2)

**FIGURE 2:**
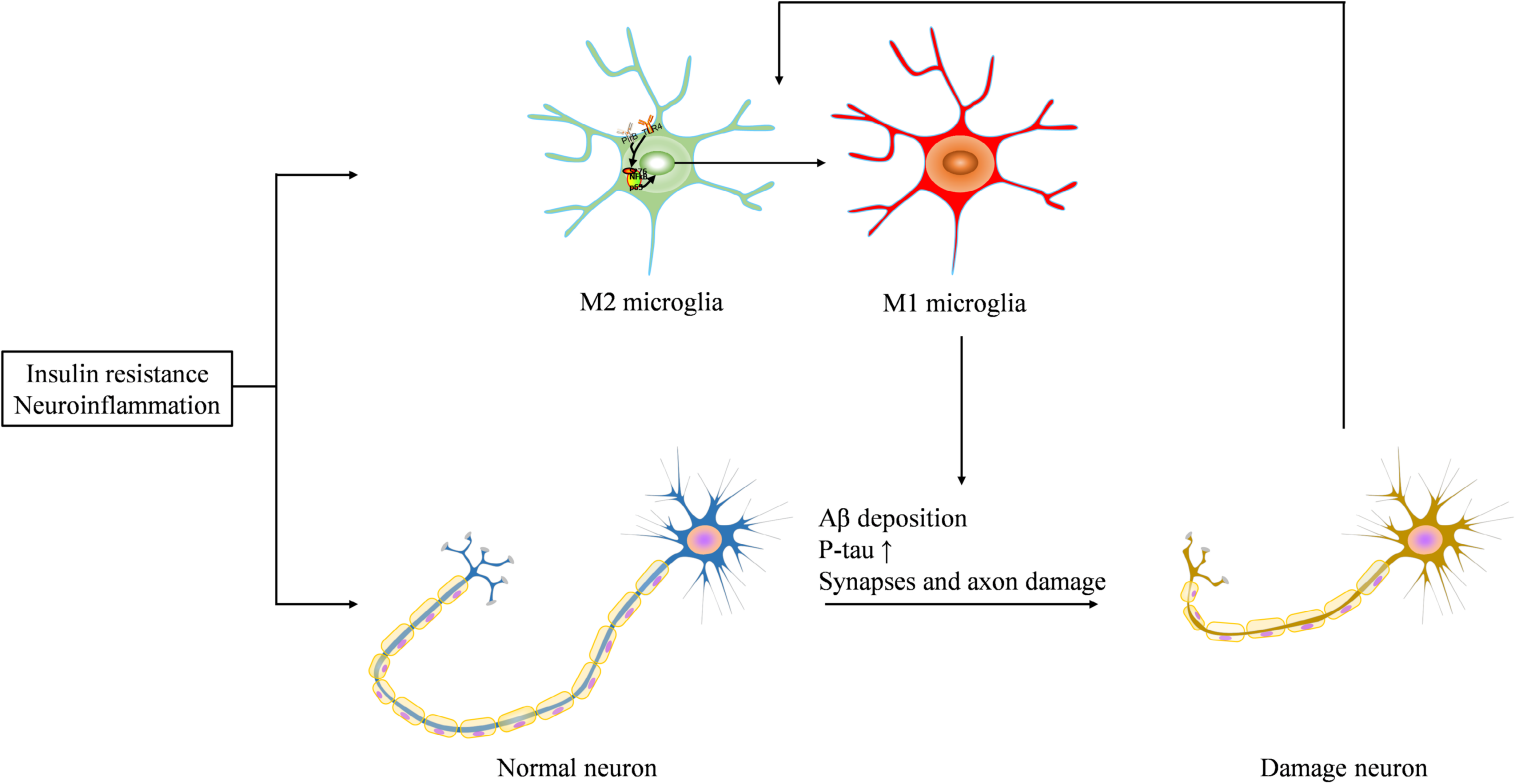
Pathological changes of diabetes‐associated cognitive dysfunction. The arrows indicate causation.

### 5.1 β‐Amyloid (Aβ) Deposition

The primary pathology underlying dementia is the accumulation of Aβ; however, the Aβ hypothesis remains controversial in the context of DACD. A population‐based cohort study found that diabetic patients were at a higher risk of having a positive amyloid status [175]. Two studies showed that diabetes might contribute to cognitive dysfunction by exacerbating Aβ accumulation in the hippocampus of mice [176, 177]. This accumulation was associated with the upregulation of the receptor for advanced glycation end products (RAGE) and AGE, the downregulation of low‐density lipoprotein receptor‐related protein and vacuolar protein sorting‐associated protein 26a, or insulin resistance [178, 179, 180, 181, 182]. In diabetic rodent models, Aβ expression in the hippocampus was obviously higher than controls, and Aβ deposition was positively associated with autophagy markers, while being negatively associated with lysosome function markers [183, 184, 185]. This indicates that the autophagy‐lysosome pathway may be involved in Aβ deposition in diabetic cognitive impairment. Moreover, neuronal calcium overload is an important factor in cognitive dysfunction. Knocking out the transient receptor potential cation channel 6, which is highly correlated with intracellular Ca^2+^ levels, decreased Aβ production in T2DM mice [186]. This suggests that intracellular Ca^2+^ concentrations regulate Aβ deposition in the brain. However, a study observed that diabetes accelerated cognitive dysfunction through cerebrovascular Aβ deposition rather than brain Aβ accumulation in an Alzheimer mouse model with diabetes [187].

Antagonizing Aβ in the hippocampus of diet‐induced obesity rats could reverse cognitive deficits, highlighting the important role of Aβ in the pathophysiology of cognitive dysfunction [188]. Furthermore, the improvement of cognitive function in diabetic rodent models was accompanied by a reduction in hippocampus Aβ levels [189, 190].

However, a cross‐sectional study reported that T2DM was not associated with CSF Aβ levels regardless of whether participants had diabetes or MCI [21]. Similarly, a study in mice with T2DM and Alzheimer's disease found that cognitive impairment caused by diabetes was unlikely to be directly dependent on Aβ deposition [191]. Therefore, we suggest that further research is needed to better understand the role of Aβ in DACD.

### 5.2 Tau Protein Hyperphosphorylation

Tau is a microtubule‐associated protein that is highly abundant in the CNS [192]. Hyperphosphorylation of tau is another feature of the diabetic brain, mainly driven by the activation of GSK‐3β, which is regulated by the insulin‐PI3K‐Akt signaling pathway [193, 194]. Compared to the control group, diabetic patients and rodent models exhibited elevated levels of P‐tau in their CSF [21, 169]. Diabetes exacerbated cognitive dysfunction by increasing P‐tau in the hippocampus of mouse models [176, 177, 195]. Protein phosphatase 2A is considered the main tau phosphatase responsible for regulating tau phosphorylation, and its methylation can reduce tau phosphorylation [194, 196]. In addition, activation of the mTOR pathway also led to hyperphosphorylation of tau in the hippocampus of diabetic rodent models [197, 198]. Chronic hyperglycemia in diabetic mice induced tau hyperphosphorylation by O‐GlcNAc transferase‐involved O‐GlcNAcylation, both in vivo and in vitro, leading to DACD [199]. High glucose levels also promoted tau hyperphosphorylation in diabetic rodent models via ALKBH5‐mediated demethylation of Dgkh m^6^A [200] and downregulation of VPS26a [182], further contributing to DACD. Moreover, insulin deficiency also promoted tau hyperphosphorylation in an Alzheimer's disease mice model [180]. Improvements in cognitive function in diabetic rodent models were accompanied by a reduction in tau hyperphosphorylation, suggesting that tau hyperphosphorylation is a critical pathological change in DACD [201, 202, 203]. However, some observational studies and diabetic animal model studies argue that P‐tau might not play a major role in the negative impact of diabetes on cognitive performance [204, 205, 206, 207]. Therefore, we recommend that further research is needed to clarify the role of tau hyperphosphorylation in DACD.

### 5.3 Synapses and Dendritic Spines Damage

Synaptic plasticity, which refers to the activity‐dependent changes in strength of neuronal connections and the modification of synaptic transmission, is a critical element in learning and memory [208]. Impairments in synaptic plasticity contribute significantly to DACD. These impairments are often assessed by long‐term potentiation (LTP) and various markers of synapses and axons, such as SNAP‐25, synaptophysin (SYP), postsynaptic density protein 95 (PSD 95), GAP‐43, and NfL [16, 209, 210]. Diabetic conditions can aggravate synaptic loss, leading to cognitive impairment [177, 211, 212], potentially through the enhancement of the interaction between F1F0 ATP synthase and Cyclophilin D [213, 214]. In diabetic rodent models with cognitive dysfunction, the number of synapses, the length of postsynaptic densities, and dendritic spine density were reduced in the hippocampus, along with axon growth and synaptic proteins such as GAP 43, SYP, and PSD 95. High glucose exposure reduced the length of the longest neurite as well as the axon initial segment [215, 216, 217]. High glucose also contributed to synaptic protein loss via impairing thrombospondin‐1 secretion from astrocytes [218]. A study in diabetic mouse models found that elevated tau levels exacerbated synaptic impairments in T1DM [195]. Some diabetes medications, while improving cognitive dysfunction, also enhance synaptic dysfunction by upregulating synaptic proteins including PSD 95, brain‐derived neurotrophic factor (BDNF), SYP, and synapsin‐1 (SYN1) in DACD rodent models [219, 220, 221]. We conclude that these findings reveal the function of synaptic damage in the development of DACD.

### 5.4 Microglia Proinflammatory Polarization

Microglia, the resident macrophages of the CNS, contribute to the development of neurodegenerative diseases [222, 223]. In a diabetic rat model, knocking down microglia reduced neuroinflammation and improved cognitive function [133]. However, excessive activation of microglia damages surrounding healthy neural tissue, and the factors released by dying or damaged neurons further exacerbate chronic microglial activation, causing progressive loss of neurons [224]. Traditionally, microglia are categorized into the proinflammatory M1 phenotype, the alternatively activated or deactivated phenotype, and the immunosuppressive M2 phenotype [224, 225]. Elevated levels of proinflammatory cytokines in the diabetic brain indicate that the innate immune system, particularly activated microglia, plays a crucial role in neuronal damage in both diabetic animals and patients [102, 226]. Moreover, several factors drive microglial activation toward the proinflammatory M1 phenotype under diabetic conditions [138, 142, 227, 228]. Interventions that reduce neuroinflammation could attenuate DACD by promoting M2 polarization of microglia and inhibiting M1 polarization [229, 230].

## 6 Treatment of DACD

Currently, there are no established treatments specifically for cognitive dysfunction in diabetic patients. While general cognitive dysfunction treatments, such as cholinesterase inhibitors and N‐methyl d‐aspartate (NMDA) receptor antagonists, have shown efficacy in neurodegenerative conditions like Alzheimer's disease, their effectiveness in DACD remains uncertain [231]. In contrast, diabetes‐specific interventions—including insulin, Glucagon‐like peptide 1 receptor (GLP‐1R) agonists, Sodium‐glucose co‐transporter 2 inhibitor (SGLT2i), and metformin—target both metabolic dysregulation and neuroinflammatory pathways, potentially offering disease‐modifying benefits in DACD patients. Currently, the symptoms of DACD could be alleviated through a combination of approaches, including antidiabetic drugs, physical activity, a healthy diet, and herbal medicine (Table 1 and sTable 1).

**TABLE 1:** Studies about treatments of DACD in diabetic patients.

Drug	Reference	Design	Participants	Groups	Application of drug (dose and time)	Outcome measures	Main findings
Insulin	Plastino et al. (2010) [232]	Prospective, open label, observational study	Patients with mild‐to moderate AD associated with T2DM	Mild‐to‐moderate AD with DM, treated with oral antidiabetic medication Mild‐to‐moderate AD with DM, treated with insulin and oral antidiabetic medications	Insulin or oral antidiabetic medications for 12 months	MMSE; CGI of severity scale	Patients in the insulin group performed better on cognitive scores than those in the oral diabetes drug group. After adjustment for these risk variables, the results remained significant
Insulin	Novak et al. (2014) [233]	Randomized, double‐blind, placebo‐controlled intervention	Older DM and non‐DM adults	DM + Placebo DM + Insulin Control + Placebo Control + Insulin	Intranasal insulin (40 IU insulin mixed with 0.4 mL saline); The placebo contained an equivalent volume of sterile saline	Hopkins Verbal Learning Test‐Revised; Trail‐Making Tests A and B; Digit Span; Rey Osterrieth Complex Figure Test; MMSE; BVMT‐R; Delis Kaplan Executive Function System assessment	Intranasal insulin improved visuospatial memory in all participants. In the DM group, an increase of perfusion after insulin administration was greater in the insular cortex compared with the control group. Cognitive performance after insulin administration was related to regional vasoreactivity
Insulin	Novak et al. (2022) [234]	Randomized clinical trial: phase 2 randomized, double‐blinded trial	Patients with diabetes and controls	DM + Intranasal insulin DM + Placebo Control + Intranasal insulin Control + Placebo	Intranasal insulin 40 IU or placebo (sterile saline) once daily for 24 weeks	CANTAB; geriatric depression scale, WHODAS 2.0; Wechsler Adult Reading and Comprehension test; MRIs	Intranasal insulin had a positive effect on cognition and gait. The intranasal insulin treated T2DM patients walked faster, had increased cerebral blood flow, and decreased plasma insulin, while the control group had improved executive function and verbal memory
Insulin	Zhang et al. (2015) [235]	Randomized, double‐blind, placebo‐controlled study	Patients with diabetes and controls	DM + Insulin DM + Placebo Control + Insulin Control + Placebo	Intranasal insulin (40 IU insulin mixed with 0.4 mL saline); The placebo contained an equivalent volume of sterile saline	MMSE; HVLT; Trail‐Making Tests A and B; Digit Span; BVMT‐R; the Verbal Fluency Task; Delis Kaplan Executive Function System assessment	A single dose of intranasal insulin increased resting state functional connectivity between the hippocampus and multiple default mode network regions in elderly patients with T2DM. Intranasal insulin administration might alter functional connections between brain regions that regulate memory and complex cognitive behavior
Metformin	Liccini et al. (2016) [236]	Observational study	Population of late middle age and older patients with diabetes mellitus	50–59 60–69 70+	Metformin or insulin or sulfonylurea	RCS	Patients with diabetes taking metformin were less likely to exhibit cognitive dysfunction on the RCS
Metformin	Samaras et al. (2020) [237]	Prospective observational study	Patients with diabetes and participants without diabetes	DM + Metformin DM No‐DM	Metformin for 6 years	Rey Auditory Verbal Learning Test; Logical Memory Story A; Benton Visual Retention Test recognition; Wechsler Adult Intelligence Scale III; Digit‐Symbol‐Coding; Trail Making Test Part A; Category Fluency Test; Boston Naming Test; Block Design from the Wechsler Adult Intelligence Scale‐Revised; Letter Fluency Test; Trail Making Test Part B	Older diabetic patients treated with metformin had slower cognitive decline and a lower risk of dementia
Metformin	Orkaby et al. (2017) [238]	Retrospective cohort study	US veterans ≥ 65 years of age with type 2 diabetes who were new users of metformin or a sulfonylurea and had no dementia	Metformin Sulfonylureas	Metformin or sulfonylureas	/	Metformin was associated with a lower risk of subsequent dementia than sulfonylurea use in veterans < 75 years of age
Metformin	Sluggett et al. (2020) [239]	Nested case–control study	Population with AD diagnosed and with diabetes diagnosed ≥ 3 years before AD	Individuals With AD Individuals Without AD	Average of 2 g metformin daily	/	Long‐term and high‐dose metformin use was associated with a lower risk of incident AD in older people with diabetes
Metformin; Sulfonylureas	Hsu et al. (2010) [240]	Prospective cohort study	Population with 50 years or older and dementia free	No type 2 diabetes DM but no DM drug Sulfonylureas only Metformin only Sulfonylureas + Metformin	Metformin/sulfonylureas	/	T2DM increased the risk of dementia more than 2‐fold Sulfonylureas and metformin decreased the risk of dementia
Metformin; sulfonylureas; glitazones; insulin	Bohlken et al. (2018) [241]	Case–Control Study	Patients with T2DM who had received a first dementia diagnosis	Patients with dementia Patients without dementia	Metformin; sulfonylureas; DPP‐4i; GLP‐1R agonists; SGLT‐2i; glitazones; insulin	/	Metformin and glitazones were negatively associated with dementia, while insulin was positively associated with dementia
Metformin	Newby et al. (2022) [242]	Comparative cohort study	Patients ≥ 50 years old with diabetes who were new users of metformin or sulfonylureas	Metformin Sulfonylureas	Metformin or sulfonylurea	/	Metformin users compared with sulfonylurea users were associated with a lower risk of all‐cause dementia, AD and VD
Metformin	Shi et al. (2019) [243]	Retrospective longitudinal cohort study	Patients with ≥ 2 diagnoses of T2DM and ≥ 50 years old as of their first diagnosis of T2DM	Non‐metformin ≤ 1 year 1–2 years 2–4 years > 4 years	Metformin	/	Long‐term metformin therapy (> 2 years) was associated with lower incidence of neurodegenerative disease (including dementia, Alzheimer's disease) among elderly veterans with T2DM
Metformin; Insulin; Metformin + insulin	Liu et al. (2024) [244]	Prospective, open label, observational study	Patients with T2DM	Insulin group Metformin group Insulin with metformin group	The insulin group received subcutaneous injections of Mendong insulin three times per day (before meals), starting with a dose of 0.6 U/kg. The metformin group was administered 1.5 g of metformin hydrochloride tablets orally once a day	MMSE	Metformin and insulin combined with metformin effectively improved MCI in T2DM patients, superior to insulin monotherapy. The efficacy of metformin was found to be comparable to that of combination therapy
Metformin	Mone et al. (2023) [245]	Observational study	Patients with diabetes and hypertension	Extended‐release Metformin Women Non‐Treated Women Regular Metformin Women Extended‐release Metformin Men	500 mg extended‐release metformin, or 500 mg regular metformin for 6 months	MoCA	Extended‐release metformin improved cognitive impairment in frail older women with hypertension and diabetes
Metformin	Guo et al. (2014) [246]	Randomized double‐blind placebo‐controlled study	Patients with T2DM and depression	Metformin Placebo	Metformin for 24 weeks	WMS‐R	Chronic treatment with metformin had an antidepressant behavioral effect, and improvements in cognitive function were associated with metformin treatment outcomes
Metformin	Rosell‐Diaz et al. (2024) [247]	An observational study and a phase IV, randomized, double‐blind, parallel‐group, randomized pilot study	Patients with T2DM	No documented medical history T2DM with metformin monotherapy T2DM treated with oral hypoglycemic agents other than metformin Newly diagnosed T2D subjects on metformin monotherapy	Metformin treatment for 4 months started with an initial dose of 425 mg per day, which was gradually increased to 1700 mg during the first week	Neurocognitive tests	The beneficial effects of metformin might be mediated by changes in the composition of gut microbiota and microbial host derived co‐metabolites
Metformin	Wu et al. (2024) [248]	Retrospective population‐based cohort	Population with ≥ 66 years newly diagnosed with diabetes	Metformin monotherapy initiation No initiation of pharmacotherapy	A standard dose of metformin monotherapy	/	Early metformin initiation was not associated with incident dementia in older adults newly diagnosed with diabetes
Metformin	Moore et al. (2013) [249]	Prospective study	Patients with AD or mild cognitive impairment and those who were cognitively intact	Model not adjusted for serum vitamin B12 levels Model adjusted for serum vitamin B12 levels	Metformin/Calcium supplements	MMSE	Metformin use was associated with impaired cognitive performance. Vitamin B12 and calcium supplements may alleviate metformin‐induced vitamin B12 deficiency and were associated with better cognitive outcomes
Metformin	Nolasco‐Rosales et al. (2023) [250]	Cross‐sectional study	Patients with T2DM	Treatment with metformin Treatment without metformin	Metformin	MMSE; HAM‐D	No association was found between metformin treatment, measures of cognitive impairment, and measures of depressive symptoms. However, chronic metformin therapy, insulin use, blood sugar control, and age could all affect the results
Metformin	Wennberg et al. (2018) [251]	Prospective, observational study	Cognitively unimpaired at baseline participants with T2DM, aged 50 years and older	T2DM Diet/Exercise Metformin Other Oral Insulin Only	metformin only, insulin only, other oral agents only, and diet and exercise only	Auditory Verbal Learning Test Delayed Recall Trial; WMS‐R; Logical Memory II & Visual Reproduction II; Boston Naming Test; Category Fluency; Trail Making Test B; WAIS‐R Digit Symbol subtest; WAIS‐R Picture Completion; Block Design subtests	Metformin use, as compared to management of diabetes with other treatments, was not associated with cognitive test performance
Metformin	Ha et al. (2021) [252]	Retrospective, observational, nested case–control study	Newly diagnosed T2DM patients, dementia‐free, and aged ≥ 50 years	Cases Controls	A total prescription of metformin for 60 > cumulative defined daily dose after DM treatment onset	/	Metformin use was associated with increased odds of AD
Metformin	Kuan et al. (2017) [253]	Retrospective cohort study	Patients who were aged > 50 years, had received a new diagnosis of T2DM	Metformin cohort Nonmetformin cohort	Metformin	/	The metformin cohort had an increased risk of all‐cause dementia. Moreover, metformin exposure increased the risk of AD and VD
Metformin + DPP‐4i; Metformin + TZD; Sulfonylurea + TZD; Metformin + Sulfonylurea	Kim et al. (2021) [254]	Retrospective observational cohort study	Those who had a blood sugar level of 126 or higher at their health screening examination or T2DM were prescribed anti‐diabetes medication	Met + SU Met + DPP‐4i Met + TZD Met + AGI SU + TZD SU + AGI Other dual therapy	Metformin, sulfonylurea, DPP‐4i; TZD; Meglitinide; AGI; insulin	/	Adding TZD or DPP‐4i instead of sulfonylurea as second‐line antidiabetic treatment may be considered for delaying or preventing dementia. Also, TZD users relative to TZD non‐users on dual oral therapy were significantly associated with lower risk of various types of dementia
Sulfonylurea; thiazolidinedione; metformin	Tang et al. (2022) [255]	Prospective observational study	Patients with T2D were aged ≥ 60 years at the initiation, and were dementia‐free were identified	SU TZD MET and SU MET and TZD SU and TZD	Sulfonylurea; thiazolidinedione; metformin	/	TZD use was associated with a lower risk of dementia compared to MET use among patients with T2DM
SGLT2i; DPP4i	Mui et al. (2021) [256]	Retrospective cohort study	Patients with T2DM	SGLT2i users DPP4i users	SGLT2i or DPP4i	/	Compared with DPP4i use, SGLT2i use was associated with a lower risk of dementia, Parkinson's disease, and cerebrovascular mortality
SGLT2i; DPP4i	Abdullah et al. (2025) [257]	Retrospective cohort study	Patients with T2DM, aged 40 years or older	SGLT2i DPP4i	SGLT2i or DPP4i	MMSE; Addenbrooke's cognitive examination; MoCA	No association was found between the use of SGLT‐2i and the risk of dementia in people with type 2 diabetes aged 40 years or older. But the use of SGLT‐2is was associated with a reduced risk of dementia in people over 65
Empagliflozin; Metformin; Insulin	Mone et al. (2022) [258]	Prospective study	Patients with HFpEF and diabetes	Empagliflozin Metformin Insulin	Empagliflozin; Metformin; Insulin	MoCA	Empagliflozin, a SGLT2i, improved cognitive impairment in frail older adults with T2DM and heart failure with preserved ejection fraction
SGLT2i; DPP4i	Osman et al. (2025) [259]	Prospective observational cohort study	Patients with T2DM on metformin therapy	Healthy volunteers Patients on metformin monotherapy Patients on metformin and DPP‐4i or SGLT2i combination therapy	SGLT2i; DPP4i; Metformin	MoCA	The combination of DPP‐4i or SGLT2i with metformin enhanced cognitive function in patients with T2DM, primarily by influencing metabolic pathways rather than directly affecting blood glucose regulation, peripheral diabetic complications, or systemic inflammation
Dulaglutide	Cukierman‐Yaffe et al. (2020) [260]	Randomized, double‐blind placebo‐controlled trial	Men and women (aged ≥ 50 years) with either established or newly diagnosed type 2 diabetes	Dulaglutide Placebo	Subcutaneous injections once a week of either dulaglutide (1.5 mg)	MoCA; DSST	After post hoc adjustment for individual standardized baseline scores, the hazard of substantive cognitive impairment was reduced by 14% in those assigned dulaglutide
Liraglutide	Li et al. (2021) [261]	Prospective study	Patients with type 2 diabetes mellitus aged 18 to 65 years with a glycated HbA1c value of > 7.0% who were treated with oral antidiabetic drugs or insulin for at least 3 months	Control Group GLP‐1 Group	Liraglutide at an initial dose of 0.6 mg/day and a maximum dose of 1.8 mg/day adjusted once a week when hyperglycemia was uncontrolled for 12‐week	MMSE	Liraglutide significantly increased activation of the dorsolateral prefrontal cortex and orbitofrontal cortex brain regions
Liraglutide	Cheng et al. (2022) [262]	Randomized parallel comparative study	Patients with type 2 diabetes inadequately controlled with metformin monotherapy	Liraglutide Dapagliflozin Acarbose	Liraglutide, dapagliflozin, or acarbose for 16 weeks. Liraglutide was gradually titrated from 0.6 mg to 1.8 mg once daily, while dapagliflozin was administered at 10 mg once daily, and acarbose was gradually increased from 50 mg to 100 mg three times daily orally with meals	MMSE; MoCA; RBANS; Trail Making Test (parts A and B); Stroop Color‐Word Test (parts I, II, and III)	Liraglutide improved disrupted brain activation and restored impaired cognitive functions in patients with type 2 diabetes, while dapagliflozin and acarbose showed no such effects
DPP4i	Jeong et al. (2021) [263]	Retrospective cross‐sectional study	Diabetic patients being treated with (ADCI‐DPP‐4i^+^ group) or without DPP‐4i (ADCI‐DPP‐4i^−^ group) and nondiabetic patients	Nondiabetic ADCI ADCI‐DPP‐4i^−^ ADCI‐DPP‐4i^+^	DPP4i	MMSE	The use of DPP‐4 inhibitors was associated with a lower amyloid burden and improved long‐term cognitive outcomes in diabetic patients with Alzheimer's disease‐related cognitive impairment
Vildagliptin + Metformin	Borzi et al. (2019) [264]	Retrospective study	Inclusion criteria were age > 65 years, MMSE score ≥ 18 and ≤ 23 and diagnosis of diabetes mellitus treated with metformin only, at a dosage of 1 g twice a day	Metformin Metformin + Vildagliptin	Vildagliptin 50 mg twice a day, metformin 1 g twice a day	MMSE	Vildagliptin in addition to metformin resulted in the maintenance of MMSE score, showing a protecting role on cognitive functioning compared to the metformin only group
Vildagliptin; sitagliptin; saxagliptin	Rizzo et al. (2014) [265]	Retrospective longitudinal study	Older patients with type 2 diabetes	DPP‐4I Group SU Group	DPP‐4I (vildagliptin 50 mg two times a day or sitagliptin 100 mg/day or saxagliptin 5 mg/day) in add on with metformin 1700 mg/day; SU (glimepiride 2 mg/day or glyburide 15 mg/day or glipizide 10 mg/day) in add on with metformin 1700 mg/day	MMSE; Trail Making Test (TMT A and B); Wechsler Adult Scale–Revised; Digit Span; Verbal Fluency Test	In older patients with type 2 diabetes affected by mild cognitive impairment, DPP‐4I improved glucose control and protected against worsening in cognitive function
DPP4i; metformin	Wu et al. (2020) [266]	Observational study	Participants using an antidiabetic medication were included in the study	Normal cognition Amnestic MCI AD dementia	Metformin; sulfonylureas; thiazolidinediones; DPP4i	MMSE	In individuals with NC, metformin use was linked to improved memory performance over time, while in those with AD, DPP‐4i use was associated with a slower rate of memory decline. Additionally, interaction analyses indicated that the cognitive benefits of DPP‐4 inhibitors were more pronounced in APOE ε4 carriers
DPP4i; GLP‐1A	Battini et al. (2024) [267]	Register‐based study	Older patients with T2DM	GLP‐1a DPP4i	DPP4i; GLP‐1A	/	Among older patients with type 2 diabetes, the use of DPP‐4 inhibitors was linked to a higher risk of developing major cognitive impairment compared to GLP‐1 receptor agonists
Linagliptin; glimepiride	Biessels et al. (2021) [268]	Randomized double‐blind study	Patients with T2DM	Linagliptin Glimepiride	Linagliptin 5 mg; Glimepiride was initiated at 1 mg and up‐titrated every 4 weeks during the first 16 weeks to a maximal dose of 4 mg	MMSE; VFT; Trail Making Test	Over a median follow‐up of 6.12 years, linagliptin (5 mg) and glimepiride (1–4 mg) showed no significant difference in the risk of Alzheimer's disease‐related cognitive decline in patients with T2DM

*Note:* All references listed in this table are included in the main reference list.

Abbreviations: AD: Alzheimer's disease; AGI: α‐glucosidase inhibitor; BVMT‐R: Brief Visuospatial Memory Test‐Revised; CANTAB: Cambridge Cognition computerized system; CGI: Clinician's Global Impression; DM: Diabetes mellitus; DPP‐4i: Dipeptidyl peptidase‐4 inhibitors; DSST: Digit Symbol Substitution Test; GLP‐1A: GLP‐1R agonists; HAM‐D: Hamilton Depression Rating Scale; HbA1c: Hemoglobin; HVLT: Hopkins Verbal Learning Test; MET: Metformin; MMSE: Mini‐Mental State Examination; MoCA: Montreal Cognitive Assessment; MRIs: Magnetic resonance imaging; NC: Normal cognition; RBANS: Repeatable Battery for the Assessment of Neuropsychological Status; RCS: Rapid Cognitive Screen; SGLT2i: Sodium‐glucose co‐transporter 2 inhibitor; SU: Sulfonylurea; T2DM: Type 2 diabetes mellitus; TZD: Thiazolidinedione; VD: Vascular dementia; VFT: Verbal Fluency Test; WHODAS: World Health Organization Disability Assessment Schedule; WMS‐R: Wechsler Memory Scale–Revised.

### 6.1 Antidiabetic Drugs

#### 6.1.1 Insulin

Insulin, the most widely used treatment for T1DM, has been increasingly utilized to manage blood glucose levels and prevent chronic complications [269]. In a prospective, open‐label, observational study, patients with T2DM in the insulin group performed better on cognitive scores than those in the oral diabetes drug group [232]. Studies based on DACD rodents found that insulin had a beneficial effect on learning and memory in diabetic models [270, 271, 272]. Insulin was found to prevent the binding of Aβ‐derived diffusible ligands (ADDL) to neurons, protect against ADDL‐induced oxidative stress, and reduce synapse loss caused by ADDL [273]. Moreover, insulin inhibits Aβ formation and accumulation in neurons [274]. Although there is evidence suggesting that insulin positively impacts cognitive function, its peripherally administered form has limited access to the CNS, requiring high doses to treat cognitive dysfunction, which increases the risk of hypoglycemic events [269]. Recently, a randomized, double‐blind, placebo‐controlled intervention found intranasal insulin improved visuospatial memory in participants with or without diabetes. In the diabetic group, perfusion in the islet cortex was greater after insulin administration compared to the control group, suggesting cognitive performance after insulin administration was associated with regional vasoreactivity [233]. A RCT reported that intranasal insulin treated T2DM patients who walked faster, had increased cerebral blood flow, and decreased plasma insulin, while the control group had improved executive function and verbal memory [234]. Moreover, a randomized, double‐blind, placebo‐controlled study found that a single dose of intranasal insulin increased resting state functional connectivity between the hippocampus and multiple default mode network regions in elderly patients with T2DM [235]. However, a major limitation is the low bioavailability of the drug, which is less than 1% when administered nasally [275]. Furthermore, two other RCTs showed that no improvement in cognitive function was observed with intranasal insulin therapy in participants with MCI or AD, including diabetic patients [276, 277]. Therefore, we recommend that subsequent RCTs further investigate the efficacy of intranasal insulin in improving DACD.

#### 6.1.2 Metformin

Metformin is the first‐line medication used to treat T2DM by controlling postprandial blood glucose levels, down‐regulating glycogen synthesis, and improving insulin sensitivity. Beyond its hypoglycemic effects, metformin has been found to protect neuronal function and enhance cognitive performance in diabetic mice [278]. Three meta‐analyses including 42 clinical trials and observational studies reported that metformin was associated with a lower risk of cognitive dysfunction in patients with diabetes [236, 237, 238, 239, 240, 241, 242, 243, 279, 280, 281]. Moreover, an observational study showed that metformin and insulin combined with metformin effectively improved MCI in T2DM patients, superior to insulin monotherapy. The efficacy of metformin was found to be comparable to that of combination therapy [244]. Another observational study showed that extended‐release metformin improved cognitive impairment in frail older women with hypertension and diabetes [245]. In a randomized double‐blind placebo‐controlled study, chronic treatment with metformin had an antidepressant behavioral effect, and improvements in cognitive function were associated with metformin treatment outcomes in patients with T2DM and depression [246]. In a population‐based study, including two independent cohorts (an observational study, and a phase IV, randomized, double‐blind, parallel‐group, randomized pilot study), the beneficial effects of metformin might be mediated by changes in the composition of gut microbiota and microbial host‐derived co‐metabolites [247]. A study in mice found that metformin improved cognitive function in diabetic mice by inhibiting mitochondrial fission, reducing mitochondrial‐derived oxidative stress, and preventing neuron loss in the hippocampus [278]. Additionally, a study in diabetic mice found that metformin alleviated diabetes‐induced cognitive impairment by enhancing autophagy to clear tau hyperphosphorylation [202]. It also reduced levels of P‐tau, Aβ, and synaptophysin in the hippocampus of diabetic mice [282]. However, some studies found negative or neutral effects when metformin was used by diabetic patients [248, 249, 250, 251, 252, 253, 283]. One cellular experiment even found that metformin increased Aβ production by up‐regulating BACE1 transcription in neurons [284]. In summary, we think the effectiveness and mechanisms by which metformin impacts DACD require further investigation.

#### 6.1.3 Thiazolidinediones (TZD)

TZD are agonists of peroxisome proliferator‐activated receptors (PPARs) that enhance insulin sensitivity and regulate glucose and lipid metabolism. A study showed that pioglitazone, a highly selective PPARγ agonist, improved memory deficits in STZ‐induced diabetic mice by reducing brain Aβ levels [190]. Moreover, in two observational studies, patients with T2DM using TZDs had an obviously lower risk of developing various types of dementia compared to those who do not use TZDs [254, 255]. We think that these findings may guide the selection of medication for patients with T2DM who are at high risk for dementia.

#### 6.1.4 SGLT2i

SGLT2i, which plays a key role in the reabsorption of urinary glucose, has been shown to improve insulin sensitivity and offer neuroprotective effects via attenuating mitochondrial dysfunction, IR, inflammation, and apoptosis in obesity [285]. A meta‐analysis including 11 clinical studies showed that the use of SGLT‐2 inhibitors was associated with a 32% lower risk of dementia compared to non‐users in patients with diabetes (HR: 0.68, 95% CI: 0.50–0.92) [286]. Other population‐based cohort studies of patients with type 2 diabetes also support the result [256, 257]. A prospective study found that empagliflozin, a SGLT2i, improved cognitive impairment in frail older adults with T2DM and heart failure with preserved ejection fraction [258]. A prospective observational cohort study reported that the combination of Dipeptidyl peptidase‐4 inhibitors (DPP‐4i) or SGLT2i with metformin enhanced cognitive function in patients with T2DM, primarily by influencing metabolic pathways rather than directly affecting blood glucose regulation, peripheral diabetic complications, or systemic inflammation [259]. Moreover, Empagliflozin mitigated cognitive impairment induced by T2DM through the modulation of oxidative stress and inflammatory pathways in hyperglycemic mice [287]. In another study, empagliflozin attenuated neurocognitive impairment through positively influencing neurochemical markers including neurotrophin levels and neuronal gene expression in diabetic mice [288]. Therefore, we suggest that SGLT2i may be a potential therapeutic approach to alleviate the progression of cognitive decline induced by T2DM.

#### 6.1.5 GLP‐1R Agonists

GLP‐1, a hormone from the incretin family, is secreted during nutrient intake and plays a crucial role in maintaining glucose metabolism. GLP‐1R agonists have both peripheral and central effects, including promoting glucose‐dependent insulin secretion, inhibiting glucagon release and gastric emptying, and inhibiting food intake [289]. As a result, their usage in treating T2DM has expanded to include obesity treatment, and recent studies have highlighted their positive effects on DACD. A RCT reported that long‐term treatment with dulaglutide could reduce cognitive impairment in individuals with T2DM [260]. Another prospective study showed that liraglutide improved cognitive decline in T2DM patients by obviously increasing activation in brain regions of the dorsolateral prefrontal cortex and orbitofrontal cortex [261]. A 16‐week randomized parallel comparative study showed that liraglutide improved disrupted brain activation and restored impaired cognitive functions in patients with type 2 diabetes [262]. Moreover, Liraglutide helped improve diabetes‐induced neuronal and synaptic damage, leading to improvements in learning and memory of diabetic mice [290, 291, 292]. In addition, Exendin‐4 (Ex‐4), a GLP‐1 analogue, alleviated learning and memory deficits in diabetic mice by mitigating tau hyperphosphorylation, increasing brain‐derived insulin levels, and enhancing the PI3K/AKT/GSK3‐β signaling pathway [203]. Overall, we think that GLP‐1R agonists show a clear potential in improving DACD.

#### 6.1.6 Dipeptidyl Peptidase‐4 Inhibitors (DPP‐4i)

Elevated DPP4 activity was identified as an independent risk factor for mild cognitive impairment in individuals with T2DM [293]. Therefore, there has been growing interest in the use of DPP‐4i to address DACD. A retrospective cross‐sectional study found that the use of DPP‐4 inhibitors was associated with a lower amyloid burden and improved long‐term cognitive outcomes in diabetic patients with Alzheimer's disease‐related cognitive impairment [263]. In other retrospective studies, vildagliptin, a DPP‐4i, was showed to protect cognitive function in diabetic patients with MCI [264, 265]. An observational study found in diabetic patients with Alzheimer's disease (AD), DPP‐4i use was associated with a slower rate of memory decline. Additionally, interaction analyses indicated that the cognitive benefits of DPP‐4 inhibitors were more pronounced in APOE ε4 carriers [266]. Among older patients with type 2 diabetes, the use of DPP‐4 inhibitors was linked to a higher risk of developing major cognitive impairment compared to GLP‐1 receptor agonists [267]. While linagliptin and glimepiride showed no significant difference in the risk of Alzheimer's disease‐related cognitive decline in patients with T2DM [268]. However, we consider that there are few studies on RCT and animal studies on DPP4i for diabetes‐related cognitive dysfunction; further research is needed to fully understand the impact of DPP4i on DACD.

#### 6.1.7 Critical Analysis of Clinical Studies and Assessment of Research Quality

Among the six drug classes reviewed, GLP‐1 receptor agonists and SGLT2 inhibitors have shown the most promising cognitive benefits in diabetic populations, supported by clinical trials indicating improvements in neuroprotection and metabolic regulation. Metformin has demonstrated potential neuroprotective effects, though findings remain inconsistent and require further validation. Insulin therapy plays a crucial role in glycemic control, but its cognitive effects vary based on treatment regimens and patient profiles. DPP‐4 inhibitors and TZDs exhibit mixed results, with some evidence suggesting reduced neuroinflammation but limited large‐scale clinical validation.

There was some heterogeneity among the clinical studies included in this review. RCTs were used in some studies, which had high causal inference ability. However, some studies are retrospective cohort studies and may be influenced by unmeasured confounding factors. Moreover, variations in study populations (age, duration of diabetes) and follow‐up time also affect the consistency of different studies. Furthermore, some RCTs were followed for short periods of time, which may have underestimated the impact of long‐term interventions. The use of different cognitive assessment tools, such as MMSE, MoCA, and ADAS‐cog, also limits the comparability of results across studies. Future research should focus on large‐scale, long‐term follow‐up RCTs to validate the sustained impact of antidiabetic drugs on cognitive function in diabetic patients. Additionally, investigating individual differences—such as APOE4 status and insulin resistance levels—will be essential for advancing personalized treatment strategies.

### 6.2 Lifestyle and Behavioral Interventions

Increasing evidence suggests that modifiable lifestyle factors play a critical role in mitigating cognitive decline in diabetic patients. These non‐pharmacological approaches can complement pharmacological treatments and may offer neuroprotective benefits.

#### 6.2.1 Dietary Interventions

High‐nutrition diets can diminish central responses to insulin, alter the gut microbiome, and activate inflammatory mediators; thus, dietary shifts and personalized therapy may offer effective interventions to prevent or improve cognitive impairment [294, 295]. A healthy diet can help regulate glucose levels, reduce oxidative stress, and improve brain function. The Mediterranean diet is Characterized by high consumption of fruits, vegetables, whole grains, nuts, olive oil, and fish. Two observational studies found this diet was associated with improved cognition in individuals with type 2 diabetes [296, 297]. In another longitudinal observational study, the Mediterranean‐DASH Intervention for Neurodegenerative Delay diet, primarily high in the intake of plant‐based food, was also associated with a slower rate of global cognition and executive function decline in older adults with type 2 diabetes [298]. Moreover, intermittent fasting protocols decreased blood glucose and improved T2DM‐induced cognitive dysfunction in rodent models [299, 300]. However, we suggest that the effect of these diets on DACD still needs to be validated in a large number of prospective clinical cohort studies.

#### 6.2.2 Physical Activity

Exercise improves glucose metabolism, increases neurotrophic factors, and reduces neuroinflammation, all of which contribute to cognitive benefits [301]. Two meta‐analyses showed that exercise intervention could improve cognitive function in patients with T2DM [302, 303]. Moreover, in another meta‐analysis, the combination of aerobic and resistance exercise could effectively improve the cognitive ability, metabolic health, and physical function of middle‐aged and elderly people with T2DM [304]. Additionally, studies found that exercise could partially reverse DACD by reducing oxidative stress and inflammation [217, 305, 306, 307, 308], or improving neuronal damage [309, 310] in the brains of T2DM animals.

Additionally, the link between T2DM and cognitive decline could be influenced by factors such as early‐life socioeconomic status, childhood cognition, and educational attainment, indicating that cognitive dysfunction in T2DM may be prevented by these aspects [311].

We conclude that a multidisciplinary approach, integrating drug, diet, exercise, cognitive training, and stress management, is likely the most effective non‐pharmacological strategy for mitigating DACD. Future research should focus on longitudinal studies to confirm the long‐term benefits of these interventions.

### 6.3 Other Drugs

Recent studies have explored the potential of various compounds in mitigating cognitive dysfunction in diabetic rodent models.

In some synthetic compounds, AB‐38b was reported to enhance cognitive function in diabetic mice by modulating oxidative stress pathways [312], while Gypenoside LXXV (GP‐75) increased the brain glucose uptake and hippocampal GLUT4 expression levels [313]. Moreover, some herbal extracts and phytochemicals, such as rolipram [128], Forsythoside B [220] and *Rosa canina* L. [314], and ginsenoside Rb1 [315] extract, showed neuroprotective effects by reducing Aβ aggregation and inhibiting hippocampal neuroinflammation in streptozotocin (STZ)‐induced diabetic rodent models. Traditional Chinese Medicines were also demonstrated potential in attenuating cognitive dysfunction in diabetic rodent models. Berberine showed to improve insulin sensitivity and reduce oxidative stress, potentially delaying diabetes‐associated cognitive decline [171, 316]. Huang‐Lian‐Jie‐Du was demonstrated antioxidative and anti‐inflammatory effects in diabetic rats, improving memory performance [317]. Jiawei Shengmai San formula was reported to reduce neuronal cell necrosis and inflammatory cell infiltration in the hippocampus in diabetic rats [318]. Zi Shen Wan Fang modulated gut microbiota and brain‐gut axis interactions, contributing to cognitive improvement in diabetic mice [319, 320]. Furthermore, treatments such as blocking voltage‐gated potassium channels [321], various inhibitors [189, 322], carnosine [164], vitamins [323, 324], fibroblast growth factor 21 [325], erythropoietin [219, 326, 327], melatonin [328, 329], and other therapeutic approaches may also play important roles in improving DACD through improving hippocampal and synaptic damage, or attenuating oxidative stress and neuroinflammation.

Although these compounds exhibit promising effects in preclinical studies, their precise mechanisms remain under investigation, and further validation through clinical trials is required before translation into human applications.

## 7 Conclusion

In this review, we have provided an overview of the diagnosis, risk factors, pathogenesis, pathological changes, and treatments related to DACD. Although important progress has been made in the study of DACD in recent years, many unresolved questions and challenges remain. First, the cognitive protective effects of different antidiabetic drugs and their underlying mechanisms have not been fully elucidated, and some study results show inconsistencies. Second, most research is still limited to animal models and cell experiments, with limited clinical trial data and few long‐term follow‐up studies. Therefore, future research should further clarify the impact of different drugs on cognitive function, optimize drug administration strategies, and explore the potential of combined therapeutic interventions. Furthermore, the development of DACD is influenced not only by biological factors but also by socioeconomic status, lifestyle, and education level, which may play crucial roles. Therefore, future research and clinical practice should emphasize personalized treatment, integrate multidisciplinary approaches, and explore precision medicine and multi‐target intervention strategies. The ultimate goal is to develop more effective cognitive protection strategies for diabetic patients, thereby reducing disease burden and improving patients' quality of life.

## Author Contributions

All authors contributed to the study conception and design. Xuefeng Yu and Xiaoyu Meng designed the study. Xiaoyu Meng, Haiyang Du, Danpei Li, Peiqiong Luo, Ranran Kan, and Yang Yan reviewed the paper and provided suggestions. Xuefeng Yu and Xiaoyu Meng wrote the paper. Xuefeng Yu is the guarantor of this work, had full access to all the data in the study, and takes responsibility for the integrity of the data. All authors reviewed the manuscript.

## Ethics Statement

The authors have nothing to report.

## Consent

The authors have nothing to report.

## Conflicts of Interest

The authors declare no conflicts of interest.

## Supporting information


**sTable 1** Studies about treatments of DACD in animal models.

## Data Availability

The authors have nothing to report.
