# Supplementary material for: Risk Factors, Pathological Changes, and Potential Treatment of Diabetes‐Associated Cognitive Dysfunction
Source: J Diabetes. 2025 Apr 28;17(4):e70089. doi: 10.1111/1753-0407.70089 (PMC12037708; doi:10.1111/1753-0407.70089)
Supplement: Supplementary file 1 — sTable 1 Studies about treatments of DACD in animal models. [file JDB-17-e70089-s001.docx]

sTable 1. Studies about treatments of DACD in animal models.

| Drug | Reference | Mouse/rat line & age or weight | Definition of the model | Application of drug (dose and method) | Experimental groups | Behavioral and molecular assessments | Main findings |
| --- | --- | --- | --- | --- | --- | --- | --- |
| Insulin | Song et al. (2018) (1) | SD rats/180-220 g | Injected one time with 50 mg/kg STZ through caudal vein | i.p. injection of 2 units insulin | (1) Control  (2) Model  (3) Treatment | MWMT | Insulin improved memory of diabetic mice, and significantly inhibited neuronal damage through Nrf2 signaling pathway. |
| Insulin | Shingo et al. (2013) (2) | Wistar rats/four-week-old/80-100 g | i.c.v. STZ to dorsal third ventricle | i.c.v. administration of insulin analogue, detemir (0.5 units=12 nmol) | (1) control  (2) STZ-3V  (3) STZ-ins | MWMT | Insulin rescued STZ-induced cognitive decline and decrease of granule cell layer neurons. |
| Insulin; glargine; metformin; glibenclamide | Chen et al. (2015) (3) | db/db mice/6 weeks old/  30-40 g | BKS.Cg-mþ/þ Leprdb/J | Metformin (200 mg/kg) and glibenclamide (10 mg/kg) was administrated orally by gavage; s.c. injection of 2 U/kg insulin glargine | (1) db/+  (2) db/db  (3) Met  (4) Gli  (5) Ins | LTP recording; MWMT; Y-maze test; OFT; | Treatment with these drugs significantly decreased hippocampal Aβ1-40 or Aβ1-42, inhibited neuronal apoptosis, and  ameliorated memory impairment. |
| Metformin | Hu et al. (2022) (4) | db^-/-^ mice/6 weeks old | Type 2 diabetic mice | Metformin (≈250 mg/kg/d) was added in drinking water for 4 weeks | (1) Control  (2) Diabetes  (3) Metformin+  Diabetes  (4) Mdivi1+ Diabetes | NORT; T-maze test | Metformin restored cognitive function by inhibiting mitochondrial fission, reducing mitochondrial-derived oxidative stress, and mitigating neuron loss in hippocampus of diabetic mice. |
| Metformin | Chen et al. (2019) (5) | db/db mice/6 weeks old | BKS.Cg-Dock7m+/+Leprdb/Nju | Metformin (200 mg/kg/d) were dissolved in saline. I.p. with metformin for 8 consecutive weeks | (1) Control  (2) db/db  (3) Met  (4) Met+CQ  (5) CQ | MWMT | Metformin attenuated cognitive impairment in db/db mice, reduced hyperphosphorylated tau proteins, restored the impaired autophagy in diabetic mice, all of which were reversed by inhibiting of autophagy activity. |
| Metformin | Li et al. (2012) (6) | db/db mice/6 weeks old | BKS.Cg-Dock7m+/+Leprdb/Nju | i.p. injection of 200 mg kg^−1^ d^−1^ metformin for 17 weeks | (1) db+  (2) db/db  (3) db/db+S  (4) db/db+M | Barnes maze | Metformin attenuated the increase of total tau, P-tau, Aβ levels, and reduction of  synaptophysin in the hippocampus of db/db mice. Metformin did not attenuate the impairments of spatial learning and memory in the db/db mice. |
| Metformin | Hwang et al. (2010) (7) | ZDF rat | A model of type 2 diabetes | Metformin was  administered orally to rats with 300 mg/kg dosage for 2 weeks. | (1) ZLC  (2) ZDF-vehicle  (3) ZDF-metformin | / | Metformin treatment normalized the reduction of cell proliferation and neuroblast differentiation in the subgranular zone of the hippocampal dentate gyrus in diabetic rats. |
| Metformin | Pintana et al. (2012) (8) | Wistar rats/5 weeks old/180 to 200 g | HFD for 12 weeks | Metformin (15 mg/kg BW twice daily) via gavage feeding for 21 days | (1) NDV  (2) NDM  (3) HFV  (4) HFM | OFT; MWMT | In the HFD group, metformin significantly attenuated the insulin resistant condition by improving metabolic parameters, decreasing peripheral and brain oxidative stress levels, and improving learning behavior. |
| Metformin; GLP-1 receptor agonist | Lennox et al. (2014) (9) | NIH Swiss mice/6-8 weeks old | HFD for 150 days. Obesity and diabetes were clearly manifested in high fat fed mice | Twice-daily s.c. injections of (Val8) GLP-1 (GluPAL)  30 nmol/kg bw; metformin (300 mg/kg bw) administered through drinking water; combined treatment with (Val8) GLP-1 (GluPAL) and metformin for 20 days | (1) HF Control  (2) HF Metformin  (3) HF (Val8) GLP-1 (GluPAL)  (4) HF Combination  (5) Lean Control | LTP; OFT; NORT; | (Val8) GLP-1 (GluPAL) treatment alone, or in combination with metformin, improved the recognition index in high fat mice, indicating enhanced learning and memory. |
| Berberine; vitamin C; metformin; donepezil | Bhutada et al. (2011) (10) | Wistar rats/200–225 g | STZ was administered at the dose of 60 mg/kg through i.p. route | Administered orally with berberine (25, 50, or 100 mg/kg), vitamin C (100 mg/kg), metformin  (500 mg/kg), or vehicle (1 ml/kg) twice daily for next 30 days | (1) ND Control  (2) ND+Ber 100  (3) D Control  (4) D+Ber 25  (5) D+Ber 50  (6) D+Ber 100  (7) D+Vit C  (8) D+Met | MWMT; Memory consolidation test; OFT | Chronic treatment with berberine, vitamin C, metformin, and donepezil during training trials improved diabetes-induced memory impairment and reduced oxidative stress and/or choline esterase activity in diabetic rats. |
| Metformin; D-allulose | Pratchayasakul et al. (2022) (11) | Wistar rats | HFD for 24 weeks | D-allulose (1.9 g/kg/day) or metformin (300 mg/kg/day) for 12 weeks | (1) NDS  (2) HFS  (3) HFR  (4) HFM | LTD and LTP; OFT; MWMT | Metformin had beneficial effects on the enhancement of brain function and cognition in prediabetic condition, and conferred greater advantage on the amelioration of brain mitochondrial dysfunction and brain microglial hyper-activation than D-allulose. |
| Metformin | Zhang et al. (2021) (12) | C57BL/6 J mice | Mice were administrated with single dose of STZ at the dose of 50 mg/kg. Then the diabetic mice were placed in an anesthetic chamber pre-filled with 98.5% O2 containing 1.5% isoflurane (1.5 L/min, 2 h) for isoflurane exposure | Mice received  metformin intragastric administration with 50 or 250 mg/kg/day for 14 days | (1) Normal  (2) Saline  (3) Metformin (50 mg/kg)  (4) Metformin (250 mg/kg) | MWMT | Metformin improved the isoflurane- and STZ-induced cognitive impairment in diabetic  mice via improving oxidative stress and inhibiting the AGEs/RAGE/NF-κB signaling pathway. |
| Metformin | Rabieipoor et al. (2023) (13) | C57BL/6 mice/ 5–6-month-old | Mice were treated with i.c.v. injections of STZ in a CSF (0.5 mg/kg) at the first and third day of experiment | Metformin (MET, 200 mg/kg per day) was used for two weeks | (1) Control  (2) STZ  (3) STZ+MET | Barnes maze test; NORT | Metformin decreased inflammatory cells and reactive astrocytes as well as the dying neurons in the hippocampus region and the cortex in SAD, and improved the cognitive performance. |
| Pioglitazone | Liu et al. (2013) (14) | ICR male mice/8–10 weeks old/20–25 g | The STZ-induced  hyperglycemic state as an animal model of T1DM by a single-  tail vein injection of STZ, 150 mg/kg body weight | Pioglitazone (18 mg/kg) was oral administration for 6 weeks | (1) Veh+Veh  (2) Veh+Pio 18 | PA test; MWMT | Pioglitazone treatment significantly ameliorated the memory deficits and  Aβ of STZ-induced diabetic mice, and activated PPARγ  in the hippocampus and cortex. |
| Empagliflozin | Khan et al. (2021) (15) | CLB57/6 mice/ 3–4 weeks/20–30g | HFruD for 7 weeks | EMPA and formulated EMPA Nps with dose of 4.4 mg/kg/day orally, respectively, for four weeks | (1) SPD  (2) HfruD  (3) HfruD + RVS  (4) HfruD + EMPA  (5) HfruD + EMPA Nps | PA test; MWMT | EMPA Nps significantly showed reduced levels of inflammatory mediators, oxidative stress, levels of p-tau, Aβ (1–40) and Aβ (1–42). |
| Empagliflozin; dapagliflozin | Piatkowska-Chmiel et al. (2023) (16) | CD-1 male mice/seven weeks old/22–25 g | Diabetes was induced for 4 weeks by ad libitum administration of 20% aqueous fructose solution to mice, followed by injection of freshly prepared STZ solution (40 mg/kg body weight, i.p.) for 5 consecutive days | EMP or DAP (10 mg/kg/day, po) for 14 days | (1) CTL  (2) DM  (3) DM-EMP  (4) DM-DAP | PA test; NORT | SGLT2i attenuated the neurocognitive impairment through the restoration of neurotrophin levels, modulation of neuroinflammatory signaling, and gene expression of Snca, Bdnf, and App in the brain of diabetic mice. |
| Liraglutide | An et al. (2021) (17) | Male diabetic db/db mice/ 4 weeks | BKS.Cg-Dock7m+/+Leprdb/Nju | LIRA diluent (200 μg/kg/d) by i.p.  injection for 5 weeks | (1) db/m  (2) db/db  (3) LIRA | MWMT; Y maze | LIRA reduced oxidative stress, lipid peroxidation and iron overload in diabetic cognitive disorders, and inhibited ferroptosis, thereby weakening the damage to hippocampal neurons and synaptic plasticity and  ultimately restoring cognitive function. |
| Liraglutide | Zhang et al. (2021) (18) | Male C57BL/6 mice/Six-week-old/18-22 g | After 8 weeks of ordinary or HFD administration, HFD mice were received i.p. injection of STZ at a dose of 60 mg/kg for consecutive 3 days after fasted for 12 h to induce diabetes | s.c. injection of liraglutide at a dose of 300 mg/kg/day for 6 weeks | (1) Control  (2) T2DM  (3) Liraglutide | MWMT | Liraglutide ameliorated DACD by rescuing autophagic flux, improved the damage of hippocampal and synaptic ultrastructure, and decreased the  accumulation of Aβ protein in hippocampus of T2DM mice. |
| Liraglutide | Yuan et al. (2019) (19) | Male C57BL/6J mice/16–20 g | Mice were i.p. injected with 65 mg/kg body weight STZ for consecutive 5 days to establish T1DM model after fasting for 12 h. | s.c. injection of liraglutide  at a dose of 250 μg/kg body weight for 6 weeks. | (1) Control  (2) Model  (3) Liraglutide | MWMT | liraglutide reversed diabetes-induced impaired learning and memory, ultrastructure damage of hippocampal neurons and synapses, exacerbated oxidative stress and neuronal apoptosis. |
| Exendin-4 | Knag et al. (2023) (20) | Male C57BL/6 mice/8-week-old; diabetic db/db mice | C57BL/6 mice: after 12 weeks feeding, the mice fed with HFD were i.p. injection with STZ at 30 mg/kg for three consecutive days; db/db mice  (C57BLKS/J-LepRdb/LepRdb) | Ex-4 at 10 μl /kg was s.c. injected into the mice | (1) WT  (2) HF-diabetic or db/db  (3) HF-diabetic+Ex-4 or db/db + Ex-4 | NORT; MWMT | Ex-4 alleviated the tau hyperphosphorylation, increased the brain-derived insulin, and improved the PI3K/AKT/GSK3-β signaling in db/db mice, HF-diabetic mice. |
| Omarigliptin | Li et al. (2022) (21) | Male C57BL/6 mice/12-week-old | Diabetic model group, 50 mg/kg STZ in 0.5 ml saline i.p. daily for five days | Omarigliptin was orally administered with 2.5 or 5 mg/kg/week for 8 weeks | (1) Sham  (2) STZ  (3) STZ + Omar 2.5 mg/kg  (4) STZ + Omar 5 mg/kg | Y-maze test | Omarigliptin ameliorated cognitive dysfunction in STZ-induced diabetic mice, and presented a protective role in the brain, as shown by the decreased ROS level, increased NAD+/NADH ratio, ATP level, and ATP synthase activity in the hippocampus. |
| Glibenclamide | Esmaeili et al. (2020) (22) | Male Wistar rats/4 months of age/180–200 g | After 2 weeks of HFD, the diabetic rats were subjected to an overnight fast and received a single i.p. injection of 35 mg/kg STZ | Rats received GBC 10 mg/kg once daily for consecutive 23 days by oral gavage | (1) CON  (2) Diabetes + Water  (3) Diabetes + GBC | Y-maze test; MWMT | GBC treatment improved memory impairment, increased insulin, and reduced glucose and hippocampal inflammation in rats with T2DM. |
| AB-38b | Chen et al. (2019) (23) | C57BL/6 mice | C57BL/6 mice  fed with HFD for two months were administrated a single i.p. injection of STZ at a dose of 100 mg/kg | AB-38b (0, 10, 20, 40 mg/kg) or resveratrol (Res,40 mg/kg) administration by gavage for 8 weeks | (1) Normal  (2) N + AB-H  (3) DM  (4) DM + AB-L  (5) DM + AB-M  (6) DM + AB-H  (7) DM + Res | NORT | AB-38b markedly increased the preference index to novel object and the number of neurons in hippocampal CA1 area of diabetic mice. Moreover, AB-38b raised Nrf2, expression and phosphorylation, as well as the protein expression and enzymatic activity of γ-glutamylcysteine synthetase, in hippocampus of the diabetic mice. |
| Gypenoside LXXV (GP-75) | Meng et al. (2022) (24) | db/db mice | C57BLKS/J-LepRdb/LepRdb | GP-75(40 mg/kg/day), and GW9662 (5 mg/kg/day) intragastrical administration for 12 weeks consecutively | (1) WT+vehicle  (2) WT+GP-75 (40mg/kg)  (3) WT+GW9662+GP-75  (4) db/db+vehicle  (5) db/db+ GP-75 (40mg/kg)  (6) db/db+ GW9662+GP-75 | MWMT | GP-75 ameliorated cognitive deficit by enhancing brain glucose uptake via the activation of Akt/GLUT4 signaling in db/db mice. |
| Astaxanthin-s-allyl cysteine diester | Loganathan et al. (2021) (25) | Male SD rats/180−200 g | The rats were fasted for 18 h and DM was induced by single i.p. injection of 45 mg/kg b.w. of STZ | AST-SAC 1 mg/kg b.w. solubilized in 1 ml distilled water administered orally for 45 days | (1) Control  (2) HG  (3) HG+5 μM AST-SAC  (4) HG+10 μM AST-SAC  (5) HG+15 μM AST-SAC | NOLT | AST-SAC administration alleviated the DM-induced injury in brain such as increased cholinesterases activity, elevated oxidative stress and mitochondrial dysfunction. |
| Dihydromyricetin | Ling et al. (2018) (26) | Male C57BL/6J mice/4weeks/ 18 ± 2 g | Mice were fed with a high-sugar and HFD for 8 weeks. After being fasted for 12 h, mice received an i.p. injection of STZ at a dose of 100 mg/kg for 3 days | DHM 125 or 250 mg/kg/d by gavage for 16 weeks | (1) Con  (2) T2DM  (3) L-DHM  (4) H-DHM | Y-maze test; MWMT | DHM significantly ameliorated cognitive impairment and reversed aberrant glucose and lipid metabolism in T2DM mice, likely through the suppression of oxidative stress and enhancement of BDNF-mediated  neuroprotection. |
| DL-3-n-Butylphthalide | Wang et al. (2021) (27) | Male db/db mice/12-week-old | C57BLKS/J-leprdb/leprdb | DL-NBP (20-120 mg/kg body weight) was administered by oral gavage and mice were treated once daily for 8 weeks | (1) db/m  (2) db/db  (3) db/db+LDL-NBP  (4) db/db+MDL-NBP  (5) db/db+HDL-NBP | MWMT | DL-NBP significantly ameliorated the cognitive decline with improved learning and memory function. Furthermore, DL-NBP administration attenuated diabetes-induced morphological alterations and increased neuronal survival and restored the levels of synaptic protein PSD95, synaptophysin and synapsin-1 as well as dendritic density in the hippocampus. |
| Ginsenoside compound K | Li et al. (2020) (28) | Female db/db mice/ 7-week-old | C57BLKS/J-leprdb/leprdb | CK group was orally administered with 10 mg kg^−1^ CK once per day for 12 weeks | (1) db/m  (2) db/db  (3) db/db+CK | MWMT; Y-maze test; Fear conditioning test | CK improved memory and cognitive dysfunction, possibly by ameliorating glucose tolerance, insulin sensitivity, and dyslipidemia, suppressing oxidative stress and inflammatory  response and modulating the NLRP3 inflammasome pathway and ER stress. |
| Caffeic acid | Castro et al. (2023) (29) | Male Wistar rats/200–250 g | Diabetes was induced by a single i.p. injection of STZ at a dose of 55 mg/kg after 6 h of fasting | CA (10 mg/kg or 50 mg/kg) was administered daily via gavage for animals | (1) Control/vehicle  (2) Control + CA 10 mg/kg  (3) Control + CA 50 mg/kg  (4) Diabetic/vehicle  (5) Diabetic + CA 10 mg/kg  (6) Diabetic + CA 50 mg/kg | OFT; NORT | CA treatment improved learning and memory deficits in diabetic rats by positive modifying the activities of cholinergic and purinergic enzymes and the density of receptors, and improving the inflammatory parameters of diabetic animals. |
| Astaxanthin | Feng et al. (2018) (30) | Male Wistar rats/140–160 g | The aT2DM group was fed a common diet for 6 weeks and then was given a high-fat, high-sugar diet feed for another 6 weeks. Then, the rats were injected with STZ i.p. at a  dose of 35 mg/kg | AST, dissolved  in polyethylene glycol 400, was administered i.p. at a dose of 25 mg/kg, three times a week for 6 weeks | (1) Control  (2) aT2dm  (3) cT2dm  (4) AST | MWMT | AST could effectively alleviate cognitive dysfunction, and might inhibit oxidative stress and inflammatory responses by activating the Nrf2-ARE signaling pathway. |
| Resveratrol | Tian et al. (2016) (31) | Male SD rats/10–12 weeks/200–250 g | A single dose of 60 mg/kg STZ was injected i.p. to induce diabetes | resveratrol (10 and 20 mg/kg/day; p.o.) from the fifth day of experiment and for 8 weeks once a day and  were administered in a constant volume of 1 ml/100 g b.w. of rat | (1) control  (2) diabetic control  (3) diabetic/RV 10 mg/kg  (4) diabetic/RV 20 mg/kg  (5) control/RV 20 mg/kg | MWMT | Chronic treatment with resveratrol improved neuronal injury and cognitive performance by attenuating oxidative stress and inflammation as well as inhibiting synapse loss in diabetic rats. |
| Resveratrol | Gocmez et al. (2019) (32) | Male Wistar rats/250–300 g | The rats in the diabetic model were induced by single dose STZ (50 mg/kg) i.p. injection in 0.1 M sodium citrate buffer | Resveratrol (20 mg/kg) was administered in a constant volume of 0.2 mL/100 g body  weight of rat | (1) Control  (2) DM  (3) DM + RSV | Locomotor activity and footshock sensitivity test; PA test; MWMT | Resveratrol treatment  improved the cognitive decline, ameliorated the impaired vascular reactivity, and significantly reversed diabetes-induced changes of protein expression. |
| Resveratrol | Hu et al. (2022) (33) | Male SD rats/6 weeks old | Rat DM models were  induced by feeding with a high-fat and high-sucrose diet for 4 weeks to develop insulin resistance, followed by 0.45% STZ (35 mg/kg) i.p. injection | RSV (i.p., 40 mg/kg) once a day for 4 weeks | (1) Control  (2) DM  (3) DM + RSV | NORT; MWMT | RSV reversed the reduced miR-146a-5p and upregulated thioredoxin-interacting protein  and inhibited the diabetes-induced increase in IL-1β and TNF-α levels in vivo and in vitro. |
| Vanillic acid | Ghaderi et al. (2023) (34) | Male Wistar rats/180–200 g | The rats were administered a single i.p. injection of freshly prepared STZ at a dose of 60 mg/kg | VA (50 mg/kg/day; P.O.) for 8 consecutive weeks | (1) Control  (2) VA  (3) STZ  (4) STZ + Ins  (5) STZ + VA | PA test; MWMT | Chronic treatment with VA alleviated blood glucose levels, improved cognitive decline, ameliorated LTP impairment, modulated oxidative-antioxidative status, inhibited inflammatory response, and prevented neuronal loss in diabetic rats at a level comparable to insulin therapy. |
| Sesamin | Farbood et al. (2019) (35) | Male Wistar rats/200 ± 20 g | Experimental diabetes was induced in rats by a single i.p. injection of STZ (60 mg/kg) | Sesamin (30 mg/kg/day; P.O.) for eight consecutive weeks | (1) Cont  (2) Ses  (3) STZ  (4) STZ + Ins  (5) STZ + Ses | MWMT | Sesamin treatment improved the spatial cognitive ability, and the expression of anti-apoptotic Bcl-2 protein in the hippocampal CA1 neurons of diabetes at a comparable level with insulin therapy. |
| Naringenin | Rahigude et al. (2012) (36) | Male SD rats /150–200 g | After two weeks of feeding with HFD, low dose of STZ (35 mg/kg) was administered | Separate groups of rats were administered with naringenin (50 mg/kg, p.o.) or vehicle (1 ml/kg, p.o.) twice daily for 58 days | (1) Control  (2) Control + Naringenin 50  (3) High fat control  (4) Diabetic control  (5) Diabetic + Naringenin 50 | NORT; OFT | Naringenin acted as an antioxidant and ChE inhibitor against type-2 diabetes-induced memory dysfunction. |
| Oleuropein | Shang et al. (2022) (37) | Male SD rats /180-220 g | The rats were fed with a high-fat and high-sugar diet for 4 weeks, and then, 35 mg/  kg STZ was i.p. injected | The rats were gavaged saline, 40 mg/kg OLE, or 30 mg/kg of SITA once a day for 4 weeks | (1) Control  (2) STZ  (3) STZ + OLE  (4) STZ + SITA | MWMT | Oleuropein ameliorated cognitive dysfunction and neuroinflammation in diabetic rats by regulating the PI3K/Akt/mTOR signaling pathway. |
| Neferine | Wu et al. (2020) (38) | Female diabetic db/db mice/7 weeks old | C57BLKS/J-leprdb/leprdb | NE groups were orally administrated NE (25 or 50 mg) dissolved in normal saline once per day for 12 weeks | (1) db/m  (2) db/db  (3) db/db + NE25  (4) db/db + NE50 | MWMT; Y-maze test; Fear conditioning test | NE treatments significantly ameliorated behavioral impairment and cognitive dysfunction in db/db mice. NE treatments also alleviated oxidative stress and inhibited inflammatory responses in the hippocampus. |
| Urolithin A | Xiao et al. (2022) (39) | Male C57BL/6J mice | After 5 weeks of feeding with HFD, mice were intraperitoneally injected with STZ at a dose of 30 mg kg–1 for 4 consecutive days | UA (200 mg kg^–1^) was intragastrically  administered for 10 weeks continuously | (1) NC  (2) T2DM + Veh  (3) T2DM + UA  (4) T2DM + Met | MWMT | Feeding UA could attenuate diabetes-associated cognitive impairment by ameliorating systemic inflammation and intestinal barrier dysfunction via N-glycan biosynthesis pathway. |
| Lycopene | Kuhad et al. (2008) (40) | Male Wistar rats/10–12 weeks/250–280 g | A single dose of 65 mg/kg STZ was injected i.p. to induce diabetes | Lycopene (1, 2 and 4 mg/kg/day; p.o.) for 10 weeks | (1) Control  (2) Diabetic  (3) Diabetic + LYCO 1  (4) Diabetic + LYCO 2  (5) Diabetic + LYCO 4  (6) LYCO 4 | MWMT | Chronic treatment with lycopene significantly and dose dependently attenuated cognitive deficit, increased acetylcholinesterase activity, oxidative–nitrosative stress and inflammation in diabetic rats. |
| Curcumin and its analog A13 | Miao et al. (2021) (41) | Male SD rats | The experimental group was fed with a HFD for four weeks. After four weeks, the diabetic model in the experimental group was established by i.p. injection of STZ (30 mg/kg) | The CUR group rats  were given curcumin 20 mg/kg. The HA group rats were intragastrically administered A13 at a dose of 20 mg/kg, and the LA group rats were 10 mg/kg | (1) NC  (2) DM  (3) CUR  (4) HA  (5) LA | / | Curcumin analog A13 could alleviate the damages in the brain of diabetes rats by regulating the pathways of inflammation and oxidative stress. |
| Fustin | Afzal et al. (2021) (42) | Male Wistar rats/200 ± 20 g | Diabetes was induced with 60 mg/kg/ip STZ | fustin treated 50 or 100 mg/kg | (1) Normal  (2) Diabetic Control  (3) Diabetic + Fustin (50 mg/kg)  (4) Diabetic + Fustin (100 mg/kg)  (5) Diabetic + Metformin (500 mg/kg) | MWMT; EPM | Fustin significant restored the behavioral parameters in treatment groups as compared to elevated levels in the diabetic control group. Furthermore, fustin significantly improved the altered levels of several biochemical parameters for cognitive dysfunction such as GSH, SOD, CAT, MDA, NO, and relative IL-6 and IL-1B compared to a diabetic control group. |
| Sesamol | Kuhad et al. (2008) (43) | Male Wistar rats/250–280 g | A single dose of 65 mg/kg streptozotocin was injected i.p.  to induce diabetes | sesamol (2, 4 and 8 mg/kg/day; p.o.) | (1) Control  (2) Diabetes  (3) Diabetes + SML  2 mg/kg/day  (4) Diabetes + SML  4 mg/kg/day  (5) Diabetes + SML  8 mg/kg/day  (6) SML 8 mg/kg/day | MWMT | Chronic treatment with sesamol significantly  and dose-dependently attenuated cognitive deficit, reduced  acetylcholinesterase, oxidative stress and inflammation in diabetic rats. |
| Paeoniflorin | Sun et al. (2017) (44) | Male SD rats/200–220 g | DM group rats were given a high-sucrose, HFD for 8 weeks. Diabetes was induced by i.p. injection of 35 mg/kg STZ | paeoniflorin (15 or 30 mg/kg), rosiglitazone (4 mg/kg) or vehicle (10 ml/kg distilled water instead) by the way of lavage once a day at 9:00–10:00 a.m. for 4 weeks | (1) Control diet  (2) DM  (3) DM + Rosiglitazone (4 mg/kg)  (4) DM + Paeoniflorin (15 mg/kg)  (5) DM + Paeoniflorin (30 mg/kg) | MWMT | Paeoniflorin had beneficial effects on relieving DACD via regulating SOCS2/IRS-1 pathway. |
| Pinocembrin | Pei et al. (2018) (45) | Adult male ICR mice /18–20 g | The ICR mice were fasted overnight and then were administered a single i.p. injection of STZ (50 mg/kg) to induce diabetes | At 24 h after induction of diabetes, Pino (50 mg/kg/day, in saline) was orally administered to mice | (1) Con  (2) DE  (3) DE + Pino  (4) DE + Vehicle | OFT; MWMT | Pino alleviated cognition deficits by protecting  neurons from inflammation injury in diabetic mice. |
| Quercetin | Hu et al. (2020) (46) | Female db/db mice/10-week-old | C57BLKS/J-leprdb/leprdb | Quercetin (35 mg/kg/day or  70 mg/kg/day) was administered for 12 weeks by gavage | (1) db/m  (2) db/db  (3) quercetin-L  (4) quercetin-H | MWMT; NORT | SIRT1/ER stress was a promising mechanism involved in quercetin-treated diabetic encephalopathy. |
| Tetramethylpyrazine | Dhaliwal et al. (2022) (47) | Male Sprague–Dawley rats /250–300 mg | After 6 weeks of HFD, a single i.p. injection of STZ (35 mg/kg) was administered to HFD fed rats | TMP (20, 40 and 80 mg/kg) was administered i.p | (1) Control  (2) DM  (3) DM + TMP (20) (4) DM + TMP (40) (5) DM + TMP (80) (6) DM + Pioglitazone (PIO, 10) | Locomotor Activity; MWMT; NO RT | Treatment with TMP alleviated learning and memory deficits, improved insulin sensitivity, and attenuated hyperglycemia and dyslipidemia in diabetic rats. Furthermore, treatment with TMP increased BDNF, p-Akt, and p-CREB levels, normalized cholinergic dysfunction, and suppressed oxidative, inflammatory, and apop-  totic markers in the hippocampus. |
| Formononetin | Wang et al. (2018) (48) | Male C57BL/6 J mice/8–10 weeks | The mice in diabetic group i.p. received a single dose of 180 mg/kg STZ | The mice were intragastrically administrated with metformin (Met, 200 mg/kg) or FMN (25,50 mg/kg) once daily for 6 weeks | (1) Control  (2) STZ  (3) STZ + metformin (Met 200 mg/kg)  (4) STZ + FMN (25 mg/kg)  (5) STZ + FMN (50 mg/kg) | MWMT; Y maze | FMN treatment effectively attenuated the body weight, learning and memory abilities, as well as the levels of blood glucose, SOD, MDA, TNF-α, IL-1β, IL-6. |
| Geniposide | Liu et al. (2019) (49) | Male ICR mice/8–10 weeks old | After 4 weeks HFD feeding, the mice in diabetic group were fasted with free access to water for 12 h and i.p. injected three times with 60 mg/kg STZ solution within 72 h | The animals were intragastrically treated  with metformin (Met, 200 mg/kg) or GEN (10, 40 mg/kg) once daily for 4 weeks | (1) Vehicel  (2) STZ + HFD  (3) STZ + HFD + Met (200 mg/kg)  (4) STZ + HFD + GEN (10 mg/kg)  (5) STZ + HFD + GEN (40 mg/kg) | Y maze; NORT; PA test; MWMT | The GEN treatment notably attenuated cognitive decline, and inhibited the generations of inflammatory cytokines. Furthermore, the administrations of GEN ameliorated the alterations of BTK, TLR4, MyD88, NF-κB, and BDNF in HFD + STZ–induced mice. |
| Rolipram | Miao et al. (2015) (50) | Male SD rats /8 weeks old/180–220 g | The diabetes and diabetes groups were given a HFD, until the end of the experiment. At the end of the 4th week, STZ was injected i.p. in HFD rats | The diabetes + rolipram group was treated with rolipram for 23 days (0.5 mg/kg, once a day, i.p.). Rolipram was injected in a volume of 1 ml/kg | (1) Control  (2) Diabetes  (3) Diabetes + ROL | MWMT | Rolipram improved cognitive function by activating the CREB signaling pathway and alleviating neuroinflammation in type 2 diabetic rats. |
| Phloridzin | Kamdi et al. (2021) (51) | Male SD rats/ ~170 g | The animals were subjected to ad libitum HFD feeding for a period of two weeks. Then, they were administered with a single i.p. injection of STZ (35 mg/kg) | Phloridzin (10 or 20 mg/kg/day, p.o.) for 28 days consecutively | (1) Normal control  (2) HFD-STZ  (3) HFD-STZ: Scopolamine (1 mg/kg, i.p.)  (4) HFD-STZ: Phloridzin (10 mg/kg)  (5) HFD-STZ: Phloridzin (20 mg/kg)  (6) HFD-STZ: Phloridzin (10) + scopolamine  (7) HFD-STZ: Phloridzin (20) + scopolamine | PA test; NORT | Reversal of T2D-induced memory impairment  by phloridzin might be attributed to upregulation of neurotrophic factors, reduced oxidative stress and increased cholinergic signaling in the brain. |
| Apocynin | Xianchu L et al. (2021) (52) | Male SD rats/about 12 weeks old/ 230 ± 20 g | Diabetic models were induced via i.p. injection of STZ (50 mg/kg) | Apocynin was orally administered at 16  mg/kg/day for 12 weeks | (1) CON  (2) DM  (3) DM + APO | MWMT | Treatment with apocynin ameliorated diabetes-related learning and memory injury. In hippocampus, apocynin markedly augmented SOD activity and inhibited MDA level to alleviate oxidative stress. |
| Asiaticoside | Yin et al. (2015) (53) | Adult male SD rats/160–180 g | Diabetes was induced by STZ (35 mg/kg, i.p.). | Separate groups of rats  were administered orally with asiaticoside (20 or 40 mg/kg), rosiglitazone (4 mg/kg), or vehicle (10 ml/kg distilled water instead) once daily for the consecutive 30 days | (1) Control  (2) DM  (3) Rosi (4 mg/kg)  (4) AS (40 mg/kg)  (5) AS (20 mg/kg) | MWMT | Asiaticoside had beneficial effects on the prevention and treatment of DACD, which was involved in oxidative stress, PI3K/Akt/NF-κB pathway and synaptic function in the development of cognitive decline induced by diabetes. |
| Forsythoside B | Nan et al. (2022) (54) | Female C57BL mice/  8-weeks-old | After anesthesia, each mouse underwent either sham operation or bilateral ovariectomy. After ovariectomy and 1 week of recovery, 60 mg/kg STZ was applied i.p. to induce diabetes | FTS·B (1 mg/kg or 150 mg/kg) and 17β-estradiol (ER) were administered i.p. once per day for 8 weeks | (1) Sham  (2) OVX  (3) DM  (4) O + D  (5) ER  (6) L-F  (7) H-F | MWMT; Y maze; NORT | FTS·B alleviated DACD by reducing the aggregation of Aβ and the hyperaggregation of p-tau in the hippocampus. Moreover, FTS·B not only inhibited inflammation by decreasing IL-1β, IL-6, and TNF-α but also modulated synaptic plasticity by increasing BDNF, PSD-95, synaptophysin, and synapsin-1. |
| Geniposide | Liu et al. (2013) (55) | Male SD rats/ aged 6 – 8 weeks | The rats were subjected to overnight fasting and i.p. injection with STZ (30 mg/kg body weight) | Geniposide treatment with intragastric administration was started on the 8th day after STZ injection and continued for 46 days (metformin was used as the positive control) | (1) Ctrl  (2) Model  (3) G12.5  (4) G25  (5) Met | / | Accompanied with the improvement of insulin and blood glucose, treatment with geniposide decreased the Aβ_1-42_ level and improved the expression of insulin-degrading enzyme, which is the key degrading enzyme of Aβ peptide. |
| Ginsenoside Rg1 | Dong et al. (2023) (56) | Male C57/BL mice/18-21 g | STZ (110 mg/kg) injections were given i.p. | The Rg1 and metformin were treated by gavage delivery for 8 weeks | (1) Control  (2) HFD  (3) HFD + STZ  (4) Rg1 (1 mg/kg)  (5) Rg1 (5 mg/kg)  (6) Rg1 (10 mg/kg)  (7) Metformin | OFT; MWMT | Rg1 therapy improved memory impairment and neuronal injury, decreased ROS, IP3, and DAG levels to revert Ca^2+^ overload, downregulated the expressions of p-PLC, TRPC6, CN, and NFAT1 nuclear translocation, and alleviated Aβ deposition in T2DM mice. In addition, Rg1 therapy elevated the expression of PSD95 and SYN in T2DM mice, which in turn improved synaptic dysfunction. |
| Hydrolea zeylanica | Swain et al. (2022) (57) | Wistar albino rats of either sex/150– 180 g | After 2 weeks of HFD, the overnight fasted HFD rats were received a single dose of STZ  (40 mg/kg, i.p.) | HHZ (300 or 400 mg/kg, p.o.) was continued for 30 days  beginning from the day of conformation of diabetes in HFD/STZ-induced rats | (1) Normal Control  (2) Negative Control  (3) Positive Control  (4) HHZ-300  (5) HHZ-400 | NORT | After 4 weeks administration of HHZ in HFD/STZ-induced diabetic rats, HHZ-400 significantly improved the learning and memory impairment with the reduction in serum glucose and elevation in insulin level in encephalopathy rats. It also significantly improved oxidative, and proinflammatory markers with the reduction in cholinesterase and β-secretase activities. |
| Hyperoside | Chen et al. (2022) (58) | Male Wistar/ six weeks old | After four weeks of HF/HFr diet, rats were deprived of food for 14 h and i.p. injected with freshly prepared STZ (35 mg/kg) | Hyperoside (50, 200, or 400 mg/kg) once daily for 6 weeks | (1) NCR  (2) DM  (3) DM + HYP50  (4) DM + HYP200  (5) DM + HYP400 | MWMT | Hyperoside prevents DM-induced cognitive dysfunction, neuroinflammation and oxidative stress via antioxidant, anti-inflammatory and antiapoptotic mechanisms in rats. |
| Luteolin | Gu et al. (2018) (59) | Male SD rats/ aged 8–10 weeks | Diabetes mellitus was induced by i.p. injection of STZ (60 mg/kg) | With the rat model established, luteolin was administered at daily doses of 50 mg/kg and 100 mg/kg by gavages in the corresponding diabetic rat groups till the end of eight weeks | (1) Control  (2) DM  (3) LT_50_  (4) LT_100_ | MWMT | Luteolin improved behavioral performances, down-regulated glycation end products in the plasma and the receptor for advanced glycation end products in the hippocampus, inhibited IL-1β and TNF-α in both the hippocampus and plasma, and upregulated the expressions of GAP-43 and SYN in the hippocampus of diabetic rats. |
| Luteolin | Liu et al. (2013) (60) | Male SD rats /10–12 weeks/200–250 g | A single dose of 60 mg/kg STZ was injected i.p. to induce diabetes | Luteolin (50 and 100 mg/kg/day, p.o.) for 8 weeks once a day | (1) Control  (2) Diabetic  (3) Diabetic + luteolin 50 mg/kg  (4) Diabetic + luteolin 100 mg/kg  (5) Luteolin 100 mg/kg | MWMT | Chronic treatment with luteolin improved neuronal injury and cognitive performance by attenuating oxidative stress and ChE activity in diabetic rats. |
| Boswellia serrate gum | Gomaa et al. (2019) (61) | Male Wistar rats/8–12 months old | Rats were fed with the HF/HFr diet. By the end of week 4, the HF/HFr-fed animals were injected with a single low dose of STZ (25 mg/kg/IP) | BS extract, orally by gavage, emulsified in 1% Tween 80 at doses of 200, 300 and 400 mg/kg. All groups received the tested drugs once daily for the rest of the 8 weeks of the experimental period | (1) NC + veh  (2) T2D + veh  (3) T2D + DON (donepezil HCl)  (3) T2D + BS200  (4) T2D + BS300  (5) T2D + BS400 | PA task; MWMT | BS extract reversed learning and memory impairment in HF/ HFr diet/STZ induced diabetic rats. This effect may be attributed to the inhibition of insulin resistance, proinflammatory cytokines, oxidative stress and hyperlipidemia. |
| Sclerocarya birrea, Nauclea latifolia, and Piper longum  mixture | Tientcheu et al. (2023) (62) | Male Wistar rats/45 to 60 days/180-200 g | T2DM was achieved by fructose feeding (10% w/v) ad libitum for 6 weeks and STZ (35 mg/kg, i.v.) to rats | Rats received p.o. the aqueous extract of SNP mixture at  the doses of 75, 150, or 300 mg/kg | (1) Normal control  (2) Diabetic control  (3) Met 200  (4) SNP 75  (5) SNP 150  (6) SNP 300 | Y‑maze test; NORT; MWMT | The aqueous extract of SNP mixture had antidiabetic and neuroprotective activities thanked to active metabolites  identified in the plant mixture, which consequently normalized blood glucose, protected hippocampus neurons, and improved memory function in diabetic rats. |
| Rosa canina L. | Ertas et al. (2023) (63) | Male SD rats/ 250 ± 20 g | In the HFD group, At the end of week 4 with HFD, diabetes was induced in HFD-fed rats with STZ after 24 h of fasting (i.p 35 mg/kg) | For four weeks, these  experimental groups were given R. canina at 250 mg/kg and MET at 400 mg/kg intragastrically per day | (1) Control  (2) HFD/STZ  (3) HFD/STZ + R.canina  (4) HFD/STZ + MET | OFT; NORT; PA test; MWMT | R. canina could correct T2DM-related cognitive decline might be attributed to insulin pathway modulation, prevention of amyloid deposition, and increased cholinergic transmission. |
| Berberine | Moghaddam et al. (2014) (64) | Male albino Wistar rats/ 225–285 g | STZ was administered at the dose of 55 mg/kg through i.p. route | Berberine chloride was administered p.o. at doses of 50 and 100 mg/kg/day 1 week after STZ injection for a period of 8 weeks | (1) Control  (2) Control + Br100  (3) Diabetic  (4) Diabetic + Br50  (5) Diabetic + Br100 | / | Chronic treatment with berberine lowered hyperglycemia, reduced oxidative stress, and  prevented the upregulation of GFAP in the brain of diabetic rats. |
| Berberine | Bhutada et al. (2011) (10) | Male Wistar rats/ 200–225 g | STZ was administered at the dose of 60 mg/kg through i.p. route | Rats were administered orally with berberine (25, 50, or 100 mg/kg) for 30 days | (1) ND Control  (2) ND + Ber100  (3) D Control  (4) D + Ber25  (5) D + Ber50  (6) D + Ber100  (7) D + Vit C  (8) D + Met | MWMT; OFT | Chronic treatment with berberine improved cognitive performance, lowered hyperglycemia, oxidative stress, and ChE activity in diabetic rats. |
| Berberine | Zhang et al. (2021) (65) | Male SD rats  /200 ± 20 g | After feeding for  eight weeks on a HFD, the rat was i.p. injected with 30 mg/kg of STZ to induce the rat diabetes model | Berberine group, with intragastrically administrated 150 mg/kg of berberine once a day for a total of consecutive four weeks | (1) Control  (2) STZ Diabetes  (3) Metformin  (4) Berberine | MWMT | In addition to improving glucose metabolism and reducing insulin resistance, berberine significantly improved the cognitive function in the rat, effectively decreased the expression of hippocampal tau protein, phosphorylated Tau, and increased insulin receptor antibodies. |
| Tangzhiqing decoction | Shi et al. (2023) (66) | Male C57BL/6J mice/6-8 weeks | After received a high-fat and high-glucose diet for 6 weeks, the model mice were given a single injection of STZ at 150 mg/kg body weight | TZQ-L 1.5g/kg, 3g/kg, or 6g/kg for eight weeks | (1) Con  (2) DM  (3) Met  (4) TZQ-L  (5) TZQ-M  (6) TZQ-H | MWMT | TZQ mitigated cognitive dysfunction in mice, while also regulating hippocampal inflammation and apoptosis. |
| Huang-Lian-Jie-Du decoction | Tian et al. (2022) (67) | Male SD rats /200 ± 20 g | Fed on a high-sugar and high-fat diet for 4 consecutive weeks later, rats fed HFD were i.p. injected with STZ (45 mg/kg body weight) | HLJDD high, medium, and low-dose groups (at 3, 1.5, and 0.75 g/kg/day termed  HLJDDH, HLJDDM, and HLJDDL, respectively) continuously for 9 weeks | (1) Control  (2) T2DM  (3) Metformin  (4) HLJDDL  (5) HLJDDM  (6) HLJDDH | MWMT | HLJDD ameliorated cognitive dysfunction in T2DM rats. The neuroprotective effect is exerted via the modulation of glucose and lipid metabolism, upregulation of autophagy, and inhibition of NLRP3 inflammasome signaling pathway. |
| Jiawei Shengmai San | Ahmed et al. (2020) (68) | Male SD rats /200–220 g | Rats were fasted for 16 hr and freshly prepared STZ (45 mg/ml) injected i.p. | J-SMS 0.5, 1.5, and 4.5 g/kg for 3 weeks | (1) Control  (2) STZ (45 mg/kg)  (3) STZ + J-SMS (0.5 g/kg)  (4) STZ + J-SMS (1.5 g/kg)  (5) STZ + J-SMS (4.5 g/kg)  (6) STZ + Donepezil (3 mg/kg) | NORT; MWMT | J-SMS treatment significantly improved learning and memory deficit among diabetic rats. Treatment with J-SMS also significantly improved the histopathological changes in the diabetic brain and increased the protein expression of AKT and CREB, required for proper memory function. |
| ZiBuPiYin recipe | Chen et al. (2017) (69) | Male C57BLKS/J-db/db mice/ 6 to 8-week-old | C57BLKS/J-leprdb/leprdb | During a period of 6 weeks, ZBPYR were administered to  the animals via gavage at a dose of 0.1 ml/10 g body weight | (1) db/m  (2) db/db  (3) db/db/ZBPYR | MWMT | ZBPYR improved DACD possibly through influencing the ‘arachidonic acid pathway’. |
| Jinzhida | Chang et al. (2013) (70) | Male SD rats/ Eight weeks-old/ 200 ± 20 g | After 4 weeks of a HFD, rats were fasted for 12 h, and each rat was injected i.p. with 30 mg/kg STZ. | Rats in the three treatment groups received different concentrations of JZD 1 ml/100 g orally twice a day for 5 weeks | (1) Cont  (2) Mod  (3) LJZD  (4) MJZD  (5) HJZD | Step-down test; MWMT | The JZD could reduce ERS and improve insulin signal transduction and insulin resistance in T2DM rats’ hippocampus and as a result improved the cognitive function. |
| Zi Shen Wan Fang | Shi et al. (2022) (71) | Male C57BL/6J mice/8 weeks-old | Mice were i.p. injected with 120 mg/kg STZ after being fed a HFD with 60% energy from fat for 3 weeks | Mice in the treatment group were orally gavaged with  crude extract of ZSWF (9.36 g/kg or 18.72 g/kg) for 8 weeks | (1) Con  (2) DCI  (3) ZSWFL  (4) ZSWFH | MWMT | ZSWF restored cognitive function in DCI mice and reduced levels of proinflammatory cytokines. Moreover, ZSWF protected the integrity of the intestinal barrier by increasing intestinal ZO-1 and occludin protein expression and decreasing urinary lactulose to mannitol ratio. |
| Zi Shen Wan Fang | Yin et al. (2022) (72) | Male C57BL/6J mice/ 12-week-old | The diabetic model was induced by i.p. injection of STZ (120 mg/kg) in the HFD group four weeks after the high fat diet | Mice were given the corresponding dosage of ZSWF (9.36 g/kg or 18.72 g/kg) by oral gavage (administration volume: mice were given 0.1 ml extract every 10 g) once a day for 8 weeks | (1) Con  (2) DCI  (3) ZSWFL  (4) ZSWFH | MWMT | ZSWF can reverse diabetes-induced cognitive impairment via activating skeletal muscle PGC1α-PPARα signaling to maintain kynurenine metabolism homeostasis. |
| voltage-gated potassium channel blockers | Yan et al. (2019) (73) | Male C57BL/6 J  mice/18–22 g | After fasted for 6 h, 65 mg/kg body weight STZ was i.p. injected for consecutive 5 days to induce diabetes | Mice were i.p. injected with TEA (5 mg/kg) or 4-AP (1 mg/kg) for 4 weeks | (1) Control  (2) Model  (3) TEA  (4) 4-AP | MWMT | TEA and 4-AP ameliorated the cognitive decline of diabetic mice, improved the ultrastructure of pancreatic β cells, hippocampal neurons and synapses, decreased oxidative stress, modulated apoptosis-related proteins, and activated PI3K/ PKB or Akt signaling pathway. |
| Calpeptin (calpain inhibitor) | Li et al. (2022) (74) | Male SD rats/ Eight-week-old/ 160 ± 10 g | The rats were fed a HFHS diet for 4 weeks. Next, the rats were i.p. administered STZ (30 mg/kg·day) for five consecutive days following 20 h of fasting. Subsequently, the rats were fed a HFHS diet for an additional 4 weeks | Rats were i.p. administered 1.75 mg/kg·day calpeptin for seven consecutive days following STZ | (1) Normal  (2) Model  (3) Calpeptin | MWMT | Calpeptin treatment significantly suppressed calpain-1 and calpain-2 expression in the hip-  pocampal tissues and effectively improved the cognitive impairments of DM model rats. Neuronal loss, Aβ accumulation, pericyte loss, inflammation, and oxidative stress injury in the hippocampal tissues of DM model rats were also partly rescued by calpeptin administration. |
| RvE1 or chemerin-9 (ChemR23 agonists) | Zhang et al. (2022) (75) | Male db/db/ 5–8 weeks of age | Leptin receptor-deficient BKS-Leprem2Cd479/Gpt mice | Mice were treated intraperitoneally with RvE1 (1.5, 3, and 6 μg/kg body weight) or C9 (30 and 60 μg/kg body weight) every other day for four weeks | (1) wt  (2) db  (3) db + RvE1  (4) db + C9 | MWMT | Administration of RvE1 or chemerin-9 ameliorated oxidative stress and inhibited NLRP3 inflammasome activation through Nrf2/TXNIP pathway, and ultimately alleviated cognitive impairment in diabetic mice. |
| Necrostatin-1 (necroptosis inhibitor) | Jinawong et al. (2020) (76) | Male rats | Rats were fed a HFD  for 13 weeks | Nec-1 (1.65 mg/kg/day s.c.) for 8 weeks | (1) NDV  (2) HFV  (3) HFM  (4) HFN | NORT; NOLT | Nec-1 and metformin equally improved cognitive function, synaptic plasticity, dendritic spine density, microglial morphology, and brain mitochondrial function and reduced  hyperphosphorylated Tau and necroptosis in HFD-fed rats. |
| PRE-084 (Sig-1R agonist) | Du et al. (2023) (77) | Male C57BL/6 mice/8–10 weeks old/ 20–25 g | Mice were i.p. injected with 50 mg/kg STZ for five consecutive days | Both PRE-084-treated  groups received i.p. PRE-084 solution at a dose of 0.25 mg/kg thrice per week from the age of 12–20 weeks | (1) CON  (2) CON + PRE-084  (3) STZ  (4) STZ + PRE-084 | Y-Maze Test; MWMT | In vivo, PRE-084 substantially reduced  ER-mitochondrion contact, activation of ERS, and C3/C3a secretion in mice with T1DM. Additionally, the synaptic loss and neurobehavioral dysfunction of mice with T1DM were less pronounced in the PRE-084 group. |
| JQ1 (BET/BRD inhibitor) | Liang et al. (2018) (78) | Male Wistar rats/200-220 g | Diabetic rat models were established by an i.p. injection of STZ (60 mg/kg body weight) after fasting for 12 h | The diabetic rats were divided into groups with i.p. injection of JQ1 (50 mg/kg) daily for twelve weeks | (1) Control  (2) DM  (3) DM + Vehicle  (4) DM + JQ1 | MWMT | Significant improvement of cognitive performance was observed after treatment with the BET/BRD inhibitor JQ1, accompanied by decreased oxidative stress, neuroinflammation and apoptosis in the hippocampus. JQ1 treatment also improved changes in the neuronal cell morphology as well as increased the expression of p-AKT, Nrf2 and HO-1. |
| Oleoylethanolamide (PPARα agonist) | Reb et al. (2019) (79) | Male C57BL/6 J mice/6–8 weeks old/ 20–22 g | Mice were fed an HFD  for 6 weeks and received an i.p. injection of STZ (40 mg/kg) | Mice received a single daily i.p. injection of OEA (15, 30 or 60 mg/kg, respectively) for 56 days | (1) Normal  (2) HFD/STZ  (3) HFD/STZ + OEA-15  (4) HFD/STZ + OEA-30  (5) HFD/STZ + OEA-60 | MWMT | Chronic OEA treatment significantly lowered hyperglycemia, recovered cognitive performance, reduced dementia markers, and inhibited hippocampal neuron loss and neuroplasticity impairments in diabetic mice. |
| Carnosine | Peng et al. (2022) (80) | db/db mice | C57BLKS/J-leprdb/leprdb | The mice were intragastrically administered with 0.9% saline or carnosine  (100 mg/kg) once daily at the age of 16 weeks and lasted  for 8 weeks | (1) db/m  (2) db/db  (3) carnosine | MWMT | Treatment with carnosine ameliorated cognitive impairment in db/db mice, reduced neuronal oxidative stress damage and iNOS expression, and relieved neurodegeneration in the hippocampus of db/db mice. |
| Carnosine | Ndolo et al. (2023) (81) | Male SD rats/8-week-old/150–160 g | Rats in the HFD group were injected i.p. with a freshly prepared solution of 1% STZ (30 mg/kg) | Rats were intragastrically administered with 100 mg/kg, 300 mg/kg, and 900 mg/kg carnosine, respectively, once a day for 82 consecutive days | (1) CON  (2) HFD/STZ  (3) CAR 100  (4) CAR 300  (5) CAR 900 | MWMT | Compared to those in the HFD/STZ group, carnosine increased SOD activity and decreased MDA levels, increased hippocampal carnosine concentration, increased p-Akt and p-mTOR expression, decreased LC3B and P62 expression, alleviated neuronal injuries, and improved cognitive performance in a dose-dependent manner. |
| Irisin | Wang et al. (2019) (82) | Male C57BL/6J  mice/ 8-week-old | A single dose of STZ (150 mg/kg) i.p. was administered to  establish a diabetic mouse model | Irisin (0.5 mg/kg/day) was given to mice through daily i.p. injection for 3 weeks | (1) Cont  (2) Irisin  (3) STZ  (4) STZ + irisin | Y Maze Test; NORT | Irisin improved memory and cognitive performance in a STZ-induced diabetic mouse model by inhibiting neuroinflammation. |
| Pyridoxamine | Kassab et al. (2019) (83) | Male Wistar  rats/ 300-400 g | Diabetes was induced with an i.p. injection of STZ (55 mg/kg in sterile saline) administered after an overnight fast | Rats were administered pyridoxamine dihydrochloride (400 mg/L or 1 g/L) in their drinking water from 1 week post-STZ | (1) Control  (2) Diabetic untreated  (3) Diabetic Pyridoxamine | NORT | Pyridoxamine had a protective effect on diabetes-induced cognitive deficits. |
| Nicotinamide riboside | Lee et al. (2019) (84) | Male ICR mice/6 weeks of age | Nicotinamide was injected to the mice assigned to HF groups at treatment week 4 to protect them from severe pancreatic beta cell damage induced by STZ. After 15 min from nicotinamide injection, STZ (100 mg/kg) was injected to the mice | NR group mice were treated with NR (400 mg/kg/day) by oral gavage daily for additional 6 weeks | (1) CON  (2) HFD  (3) NR | Nest Construction Test; Y-maze Test | NR may be useful for treating cognitive impairment by inhibiting amyloidogenesis and neuroinflammation. |
| Vitamin D and rosuvastatin | Muneeb et al. (2022) (85) | Male SD rats/ 150–180 g | At the beginning of the 5th week with a HFSD, a single subdiabetogenic dose of STZ (35 mg/kg; IP) was given after an overnight fast | The dose of VitD was 500 IU/kg/day; PO, while that for RSV was 15mg/kg/day; PO daily for 5 weeks with HFSD | (1) NC  (2) T2DM  (3) VitD  (4) RSV  (5) VitD + RSV | NORT; MWMT | VitD and/or RSV amelioratied T2DM-induced hippocampal insult and accompanied behavioral alterations. |
| Vitamin D | Hussein et al. (2022) (86) | Male Wistar rats/8 weeks old/ 150–200 g | At the end of week 6 with HFSD, rats were fasted for 8 h prior to receiving STZ (40 mg/kg, i.p.) | VD was freshly diluted in corn oil and was administered at a daily oral dose of 500 IU/kg | (1) Normal control  (2) HFSD/vehicle  (3) HFSD/STZ  (4) HFSD/STZ/  metformin  (5) HFSD/STZ/  vehicle  (6) HFSD/STZ/VD | OFT; NORT; MWMT | VD ameliorated HFSD/STZ-induced dysbiosis/gut barrier dysfunction, suppressed HFSD/STZ-induced hyperglycemia, hyperinsulinemia, dyslipidemia, and  hippocampal neuroinflammation, and improved cognitive function. |
| Erythropoietin | Yan et al. (2023) (87) | Male C57BL/6 J mice/16–18 g | The model mice were i.p. injected with 65 mg/kg body weight STZ for five consecutive days | Mice were given a  s.c. injection of EPO at a dose of 450 IU/kg body weight thice a week for 6 weeks | (1) Control  (2) T1DM  (3) T1DM + EPO | MWMT | EPO treatment alleviated STZ-induced diabetic mice showed impaired spatial learning and memory. EPO also significantly lowered elevated fasting blood glucose levels, improved pancreatic and hippocampal damage, and restored oxidative stress in the STZ-induced diabetic mice. |
| Erythropoietin | Guo et al. (2023) (88) | Male C57BL/6 J mice/18–22 g | the T2DM group was fed a HFD for 8 weeks. After fasting for 12 h, the T2DM group was administered a continuous daily i.p. injection of STZ at a dose of 60 mg/kg for 3 days | the EPO group received s.c. injections with 500 IU/kg recombinant human EPO three times a week for 6 weeks | (1) Control  (2) Model  (3) EPO | MWMT | EPO improved cognitive impairments, decreased fasting blood glucose, and ameliorated hippocampal damage in the brain of diabetic mice, which might be related to decreasing iron overload and inhibiting ferroptosis. |
| Erythropoietin | Wang et al. (2017) (89) | Male SD rats /160–180 g | The diabetic rats were fed on a high-fat, HG diet. Six weeks later, the model rats were given a peritoneal injection of freshly prepared STZ (25 mg/kg body weight) after fasting overnight. | Recombinant human erythropoietin was administered to the diabetic rats by hypodermic injection at 300 U/kg 3 times a  week for 4 weeks | (1) Control  (2) Model  (3) EPO | MWMT | EPO prevented deficits in spatial learning and memory in diabetic rats. |
| Fibroblast growth factor 1 | Wu et al. (2020) (90) | Male db/db mice/twelve-week-old | C57BLKS/J-leprdb/leprdb | The db/db mice were i.p. injected with FGF1 (0.5 mg/kg body weight) every other day for 4weeks | (1) db/m  (2) db/db  (3) db/db + FGF1 | MWMT | FGF1 significantly ameliorates DACD with better spatial learning and memory function. Moreover, FGF1 blocked diabetes-induced morphological structure change, neuronal apoptosis and Aβ1–42 deposition and synaptic dysfunction in hippocampus. |
| Fibroblast growth factor 1 | Zhao et al. (2022) (91) | Male C57BL/6 mice/six-week-old/ 18-22 g | T1D was induced using  STZ (i.p., 50 mg/kg) for five consecutive days | The diabetic mice were received FGF21 (2 mg/kg, i.p.) daily for four consecutive weeks | (1) CON  (2) DM  (3) DM/FGF21 | MWMT | FGF21 treatment improved LDH-B activity, β-nicotinamide adenine dinucleotide, and ATP levels, and increased MCT2 expression and PI3K signaling pathway, which in turn improved the learning and memory defects. |
| Hydrogen sulfide | Ma et al. (2017) (92) | Male C57BLKs-J/db-db mice/8-week-old | C57BLKS/J-leprdb/leprdb | According to body weight, NaHS solution (4.48 mg/kg), which served as an H2S donor, was injected i.p. once a day | (1) Control  (2) db/db + NaHS  (3) db/db | MWMT | H2S improved the spatial learning and memory abilities of the db/db mice by modulating the mitochondrial apoptotic pathway and the IL-23/IL-17 axis, which were found to be associated with DACD. |
| Hydrogen sulfide | Zou et al. (2017) (93) | Male SD rats /280-300 g | STZ was administered at the dose of 40 mg/kg through i.p. route | Rats were received an intraperitoneal  infusion of NaHS (100 μmol/kg/d) for 30 d | (1) Control  (2) STZ  (3) STZ + NaHS30  (4) STZ + NaHS100  (5) NaHS100 | NORT; Y- maze test; MWMT | Treatment of diabetic rats with sodium hydrosulfide (NaHS, a donor of H2S) significantly reversed diabetes-induced impairment in cognitive function. |
| Hydrogen sulfide | He et al. (2021) (94) | Male SD rats/8–10-week-old/260–300 g | In STZ-induced diabetic  group, rats were i.p. injected with freshly prepared STZ (50 mg/kg) after fasting overnight | Rats were i.p. administrated with NaHS (30 or 100 µmol/kg/day) for 30 days | (1) Control  (2) STZ  (3) STZ + NaHS30  (4) STZ + NaHS100  (5) NaHS100 | Y‑Maze Test; NORT; MWMT | SIRT1 mediated the protection of H_2_S against cognitive dysfunction in STZ-diabetic rats partly via inhibiting hippocampal ER stress and synaptic dysfunction. |
| Melatonin | Cui et al. (2021) (95) | Male C57BL/6J mice/8- week-old | The type 2 diabetic mice model was induced by injection of STZ (100 mg/kg body weight) and fed with a HFD for 16 weeks | MLT (10 mg/kg/  day) was injected i.p. once a day for 1 month | (1) CON  (2) MLT  (3) T2DM  (4) T2DM + MLT | MWMT | MLT treatment ameliorated learning and memory impairments OF type 2 diabetic mice. Meanwhile, MLT administration significantly improved neuroinflammation and regulated microglial apoptosis. |
| Melatonin | Albazal et al. (2021) (96) | Male Wistar rats/ 10–12 weeks old/ 205−250 g | To induce diabetes, rats  received i.p. injection of STZ (at a dose of 60 mg/kg) | Melatonin was  administered i.p. (10 mg/kg/day) for 7 weeks on a regular basis | (1) Control  (2) Control + Melatonin  (3) Diabetic  (4) Diabetic + Melatonin | Y-maze task; NOD test; PA test | Melatonin could ameliorate learning and memory disturbances in diabetic rats through mitigation of cholinesterase activity, astrocytes, oxidative stress and inflammation and also via upregulation of some antioxidants in addition to its prevention of dendritic spine loss. |
| Lipopolysaccharide | Mizobuchi et al. (2021) (97) | Male C57BL/6 mice/ 6 weeks of age/19-24 g | To induce type I diabetes, mice were i.p. injected with STZ at a dose of 200 mg/kg b.w. | LPS was dissolved in  drinking water at 1 mg/kg b.w./day. LPS was orally administered to mice 1 week prior to STZ injection and until the end of the experiment | (1) PBS i.p.  (2) STZ i.p.  (3) STZ i.p. + LPS | MWMT | Oral administration of LPS prevented STZ-  induced diabetic cognitive impairment, but did not affect blood glucose levels. |
| DL-3-n-butylphthalide | Gao et al. (2019) (98) | Male homozygote db/db mice/ six-week | C57BLKS/J-leprdb/  leprdb | NBP was administered to the mice in the db/db+NBP group by gavage for 6 weeks at a dose of 120 mg/kg | (1) db/m  (2) db/db  (3) db/db + NBP | MWMT | NBP ameliorated cognitive deficits induced by type 2 diabetes mellitus through improving CaMKII-mediated LTP and cell ultrastructure in the hippocampus. |
| Fingolimod | Sood et al. (2023) (99) | Male C57BL6 mice/8 weeks old | After 1 month on the HFD, the mice were given a single low dosage of STZ (100 mg/kg/i.p.) | Mice were administered fingolimod at 0.1 mg/kg, i.p., (LD), 0.5 mg/kg, i.p., (MD) and 1 mg/kg, i.p. (HD) thrice a week for 30 days | (1) NC  (2) DC  (3) LD  (4) MD  (5) HD  (6) Perse | MWMT; Object Location test; NORT; Y Maze Test | Fingolimod improved M2 polarization and synaptic plasticity while ameliorating cognitive decline and neuroinflammation. |
| Fish oil | Wang et al. (2020) (100) | Male SD rats  /160-180g | Diabetes was established with a HFD for two weeks. After overnight fasting, rats were injected i.p. with 40 mg/kg STZ | Rats were fed a  standard containing 4% FO (1.5 g/kg/d) | (1) CON  (2) CON + FO  (3) DM  (4) DM + FO | MWMT | FO administration improved spatial learning and memory, significantly improved the morphology of neurons in the hippocampus and cortex of diabetic rats and reduced the neuronal nuclear condensation. Moreover, FO attenuated the brain inflammatory cascade and simultaneously reduced diabetes-induced oxidative stress. |
| Folic acid | Zheng et al. (2017) (101) | Male C57Bl/6J mice/ 9-10-week-old | To induce diabetes in the mice, which were kept in fasted condition for 12 h prior to injection, a single i.p. injection of 150 mg STZ was administered per kg of mouse body weight | The folate-deficient diet (0.2 mg/kg folic acid diet), the control diet (2.1 mg/kg folic acid diet) and daily gavage with 120 µg/kg folic acid were administered for eight weeks | (1) NC  (2) DM-FD  (3) DM-FN  (4) DM-FS  (5) DM-Ins | / | Folic acid reduced tau hyperphosphorylation at Ser396 in the brain of diabetes mellitus mice. |
| Levetiracetam | Zhang et al. (2023) (102) | Male SD rats/ 200 ± 10 g | Rats in the STZ groups were subjected to one dose of i.p. STZ (60 mg/kg) | LEV was gavaged 10 mg/kg or 100 mg/kg for 12 weeks | (1) ctrl  (2) STZ  (3) STZ + LEV-L  (4) STZ + LEV-H | MWMT | Levetiracetam attenuates STZ-induced cognitive impairment by suppressing microglia  activation. |
| Genistein | Rajput et al. (2017) (103) | Male swiss albino mice /8 weeks or older/25-30 g | STZ was injected in mice at the dose of 200 mg/kg through i.p. route | Mice were administered genistein (2.5, 5.0 and 10.0 mg/kg, i.p.)  and metformin (500 mg/kg), vitamin C (100 mg/kg) or vehicle  (1 ml/kg) (DMSO) once daily, orally for 30 days | (1) ND control  (2) ND + Gen (10.0 mg/kg)  (3) D Control  (4) D + Gen (2.5 mg/kg)  (5) D + Gen (5.0 mg/kg)  (6) D + Gen (10.0 mg/kg)  (7) D + Vit c (100 mg/kg)  (8) D + Met (500 mg/kg) | MWMT; OFT | Chronic genistein treatment in diabetic mice significantly lowered hyperglycemia, recovered  cognitive performance by affecting acetylcholinesterase activity and oxidative stress and ameliorated neuro-inflammatory condition by varying TNF-a, IL-1β and nitrite levels as impaired in diabetic mice. |
| Quetiapine | Wang et al. (2019) (104) | Male C57BL/6J mice/ 8-week-old | Single-dose i.p. injection of STZ (150 mg/kg) was administrated to induce a diabetic mouse model | QTP (5 mg/kg/day) was given to mice in drink water 1 week before the single-dose STZ injection and lasted for 5 weeks | (1) Cont  (2) Cont + QTP  (3) STZ  (4) STZ + QTP | Puzzle Box Test | QTP effectively attenuated behavioral deficit, inhibited the activation of astrocytes and microglia in diabetic mice, and reduced the generation and release of TNF-α and MCP-1. Meanwhile, QTP also prevented the protein loss of the SYP and MBP. |
| N-Acetyl cysteine | Saha et al. (2023) (105) | Male C57/BL mice/four to  five weeks old | Mice were fed with a HFD for 5 months to induce obesity and diabetes | Mice were orally treated (using an oral gavage) with NAC (50 and 100 mg/kg) daily for 4 months initially | (1) Control  (2) High fat  (3) High fat + NAC 50 mg/kg  (4) High fat + NAC 100 mg/kg | Grip Strength; TST; OFT; NORT; EPM; Y-Maze Test; MWMT | Oral treatment with NAC decreased Cdk5 kinase activity in the hippocampus, attenuated high levels of phospho-tau (ser396), and ameliorated memory and learning impairments in a T2DM mouse model. |
| Novel adamantane derivatives | Piatkowska-Chmiel et al. (2022) (106) | Male CD-1 mice/seven-eight-week-old/20–23 g | Mice were administered 20% aqueous solution of fructose ad libidum for 4 weeks and then for the next 5 days they were administered i.p. injection of freshly prepared solution of STZ | Mice received orally 1 × daily for the next 14 days aqueous solutions of tested compounds (0.01 ml/kg body weight): adamantane derivatives: compound 2 and 3 (50 mg/kg), vildagliptin (20 mg/kg) or saxagliptin (10 mg/kg) | (1) CTL  (2) DM  (3) DM-Vil  (4) DM-Sax  (5) DM-compound2  (6) DM-compound3 | OFT; The Y-maze spontaneous alternation test; The Y maze; NPT; NORT; PA test | New adamantane derivatives, similarly to DPP4 inhibitors, could restrict diabetes‑induced cognitive deficits. |
| Transplantation of platelet-derived mitochondria | Ma et al. (2020) (107) | Homozygous male db/db mice | C57BLKS/J-leprdb/  leprdb | The animals underwent i.c.v. injection with 1× 10^5^ per 5 μl Mito-Plt suspension or isometric amount of vehicle for 10 min | (1) Control  (2) Control + mito  (3) db/db  (4) db/db + mito | MWMT | Transplantation of Mito-Plt attenuated cognitive impairment and mitochondrial  dysfunction in db/db mice. |

Abbreviations: SD: Sprague-Dawley; STZ: Streptozotocin; i.p.: Intraperitoneal; MWMT: Morris water maze test; i.c.v.: Intracerebroventricularly; s.c.: Subcutaneous; OFT: Open field test; LTP: Long-term potentiation; NORT: Novel object recognition test; ZDF: Zucker diabetic fatty; HFD: High fat diet; LTD: Long-term depression; HFruD: High fructose diet; Nps: Nanoparticles; SPD: Standard pellet diet; EMPA: Empagliflozin; PA: Passive Avoidance; SGLT2i: Sodium-glucose co-transporter 2 inhibitor; DAP: Dapagliflozin; LIRA: Liraglutide; T2DM: Type 2 diabetes mellitus; ROS: Reactive oxygen species; ATP: Adenosine triphosphate; GBC: Glibenclamide; GP-75: Gypenoside LXXV; AST-SAC: Astaxanthin-s-allyl cysteine diester; b.w.: Body weight; NOLT: Novel object location test; DHM: Dihydromyricetin; DL-NBP: DL-3-n-Butylphthalide; CK: Ginsenoside compound K; AST: Astaxanthin; VA: Vanillic acid; OLE: Oleuropein; SITA: Sitagliptin; NE: Neferine; EPM: Elevated Plus Maze; Pino: Pinocembrin; TMP: Tetramethylpyrazine; FMN: Formononetin; GEN: Geniposide; ROL: Rolipram; FTS·B: Forsythoside B; BS: Boswellia serrate; HF/HFr: High fat/high fructose; SNP: Sclerocarya birrea, Nauclea latifolia, and Piper longum mixture; TZQ: Tangzhiqing decoction; ZBPYR: ZiBuPiYin recipe; JZD: Jinzhida; ZSWF: Zi Shen Wan Fang; HFHS: High-fat and high-sugar; NR: Nicotinamide riboside; HFSD: High-fat sucrose diet; FGF1: Fibroblast growth factor 1; H2S: Hydrogen sulfide; NOD: Novel object discrimination; LPS: Lipopolysaccharide; NBP: DL-3-n-butylphthalide; LD: Low dose; MD: Mid-dose; HD: High dose; LEV: Levetiracetam; SYP: Synaptic protein synaptophysin; MBP: Myelin basic protein; NAC: N-Acetyl cysteine; TST: Tail Suspension Test; EPM: Elevated Plus Maze; NPT: Novelty preference test

Note: All references listed in this table are separate from the main reference list. Critical analysis of diabetic animal model studies: There is a lack of long-term follow-up data, as most studies are short-term experiments, making it difficult to assess the long-term cognitive protective effects. Most studies are based on STZ- or HFD-induced diabetes models, which may not fully align with the pathological mechanisms of human DACD, leading to limited translational applicability. Currently, only a few clinical trials have evaluated the cognitive improvement effects of these drugs in diabetic patients, highlighting the need for more RCTs for further validation in the future.

**References**

1. Song Y, Ding W, Bei Y, Xiao Y, Tong HD, Wang LB, Ai LY. Insulin is a potential antioxidant for diabetes-associated cognitive decline via regulating Nrf2 dependent antioxidant enzymes. Biomed Pharmacother 2018;104:474-484.

2. Shingo AS, Kanabayashi T, Kito S, Murase T. Intracerebroventricular administration of an insulin analogue recovers STZ-induced cognitive decline in rats. Behav Brain Res 2013;241:105-111.

3. Chen F, Dong RR, Zhong KL, Ghosh A, Tang SS, Long Y, Hu M, et al. Antidiabetic drugs restore abnormal transport of amyloid-beta across the blood-brain barrier and memory impairment in db/db mice. Neuropharmacology 2016;101:123-136.

4. Hu Y, Zhou Y, Yang Y, Tang H, Si Y, Chen Z, Shi Y, et al. Metformin Protects Against Diabetes-Induced Cognitive Dysfunction by Inhibiting Mitochondrial Fission Protein DRP1. Front Pharmacol 2022;13:832707.

5. Chen JL, Luo C, Pu D, Zhang GQ, Zhao YX, Sun Y, Zhao KX, et al. Metformin attenuates diabetes-induced tau hyperphosphorylation in vitro and in vivo by enhancing autophagic clearance. Exp Neurol 2019;311:44-56.

6. Li J, Deng J, Sheng W, Zuo Z. Metformin attenuates Alzheimer's disease-like neuropathology in obese, leptin-resistant mice. Pharmacol Biochem Behav 2012;101:564-574.

7. Hwang IK, Kim IY, Joo EJ, Shin JH, Choi JW, Won MH, Yoon YS, et al. Metformin normalizes type 2 diabetes-induced decrease in cell proliferation and neuroblast differentiation in the rat dentate gyrus. Neurochem Res 2010;35:645-650.

8. Pintana H, Apaijai N, Pratchayasakul W, Chattipakorn N, Chattipakorn SC. Effects of metformin on learning and memory behaviors and brain mitochondrial functions in high fat diet induced insulin resistant rats. Life Sci 2012;91:409-414.

9. Lennox R, Porter DW, Flatt PR, Holscher C, Irwin N, Gault VA. Comparison of the independent and combined effects of sub-chronic therapy with metformin and a stable GLP-1 receptor agonist on cognitive function, hippocampal synaptic plasticity and metabolic control in high-fat fed mice. Neuropharmacology 2014;86:22-30.

10. Bhutada P, Mundhada Y, Bansod K, Tawari S, Patil S, Dixit P, Umathe S, et al. Protection of cholinergic and antioxidant system contributes to the effect of berberine ameliorating memory dysfunction in rat model of streptozotocin-induced diabetes. Behav Brain Res 2011;220:30-41.

11. Pratchayasakul W, Jinawong K, Pongkan W, Jaiwongkam T, Arunsak B, Chunchai T, Tokuda M, et al. Not only metformin, but also D-allulose, alleviates metabolic disturbance and cognitive decline in prediabetic rats. Nutr Neurosci 2022;25:1115-1127.

12. Zhang W, Zhao L, Zhang J, Li P, Lv Z. Metformin improves cognitive impairment in diabetic mice induced by a combination of streptozotocin and isoflurane anesthesia. Bioengineered 2021;12:10982-10993.

13. Rabieipoor S, Zare M, Ettcheto M, Camins A, Javan M. Metformin restores cognitive dysfunction and histopathological deficits in an animal model of sporadic Alzheimer's disease. Heliyon 2023;9:e17873.

14. Liu LP, Yan TH, Jiang LY, Hu W, Hu M, Wang C, Zhang Q, et al. Pioglitazone ameliorates memory deficits in streptozotocin-induced diabetic mice by reducing brain beta-amyloid through PPARgamma activation. Acta Pharmacol Sin 2013;34:455-463.

15. Khan T, Khan S, Akhtar M, Ali J, Najmi AK. Empagliflozin nanoparticles attenuates type2 diabetes induced cognitive impairment via oxidative stress and inflammatory pathway in high fructose diet induced hyperglycemic mice. Neurochem Int 2021;150:105158.

16. Piatkowska-Chmiel I, Herbet M, Gawronska-Grzywacz M, Pawlowski K, Ostrowska-Lesko M, Dudka J. Molecular and neural roles of sodium-glucose cotransporter 2 inhibitors in alleviating neurocognitive impairment in diabetic mice. Psychopharmacology (Berl) 2023;240:983-1000.

17. An JR, Su JN, Sun GY, Wang QF, Fan YD, Jiang N, Yang YF, et al. Liraglutide Alleviates Cognitive Deficit in db/db Mice: Involvement in Oxidative Stress, Iron Overload, and Ferroptosis. Neurochem Res 2022;47:279-294.

18. Zhang M, Yan W, Yu Y, Cheng J, Yi X, Guo T, Liu N, et al. Liraglutide ameliorates diabetes-associated cognitive dysfunction via rescuing autophagic flux. J Pharmacol Sci 2021;147:234-244.

19. Yan W, Pang M, Yu Y, Gou X, Si P, Zhawatibai A, Zhang Y, et al. The neuroprotection of liraglutide on diabetic cognitive deficits is associated with improved hippocampal synapses and inhibited neuronal apoptosis. Life Sci 2019;231:116566.

20. Kang X, Wang D, Zhang L, Huang T, Liu S, Feng X, Guo Y, et al. Exendin-4 ameliorates tau hyperphosphorylation and cognitive impairment in type 2 diabetes through acting on Wnt/beta-catenin/NeuroD1 pathway. Mol Med 2023;29:118.

21. Li X, Yin Y, Li W, Li S, Zhang D, Liu Z. Omarigliptin alleviates cognitive dysfunction in Streptozotocin-induced diabetic mouse. Bioengineered 2022;13:9387-9396.

22. Esmaeili MH, Enayati M, Khabbaz Abkenar F, Ebrahimian F, Salari AA. Glibenclamide mitigates cognitive impairment and hippocampal neuroinflammation in rats with type 2 diabetes and sporadic Alzheimer-like disease. Behav Brain Res 2020;379:112359.

23. Chen YJ, Tang ZZ, Du L, Liu Y, Lu Q, Ma TF, Liu YW. A novel compound AB-38b improves diabetes-associated cognitive decline in mice via activation of Nrf2/ARE pathway. Brain Res Bull 2019;150:160-167.

24. Meng X, Zhang Y, Li Z, Hu J, Zhang D, Cao W, Li M, et al. A novel natural PPARgamma agonist, Gypenoside LXXV, ameliorates cognitive deficits by enhancing brain glucose uptake via the activation of Akt/GLUT4 signaling in db/db mice. Phytother Res 2022;36:1770-1784.

25. Loganathan C, Sakayanathan P, Thayumanavan P. Astaxanthin-s-allyl cysteine diester against high glucose-induced neuronal toxicity in vitro and diabetes-associated cognitive decline in vivo: Effect on p53, oxidative stress and mitochondrial function. Neurotoxicology 2021;86:114-124.

26. Ling H, Zhu Z, Yang J, He J, Yang S, Wu D, Feng S, et al. Dihydromyricetin improves type 2 diabetes-induced cognitive impairment via suppressing oxidative stress and enhancing brain-derived neurotrophic factor-mediated neuroprotection in mice. Acta Biochim Biophys Sin (Shanghai) 2018;50:298-306.

27. Wang BN, Wu CB, Chen ZM, Zheng PP, Liu YQ, Xiong J, Xu JY, et al. DL-3-n-butylphthalide ameliorates diabetes-associated cognitive decline by enhancing PI3K/Akt signaling and suppressing oxidative stress. Acta Pharmacol Sin 2021;42:347-360.

28. Li CW, Deng MZ, Gao ZJ, Dang YY, Zheng GD, Yang XJ, Chao YX, et al. Effects of compound K, a metabolite of ginsenosides, on memory and cognitive dysfunction in db/db mice involve the inhibition of ER stress and the NLRP3 inflammasome pathway. Food Funct 2020;11:4416-4427.

29. Castro MFV, Assmann CE, Stefanello N, Reichert KP, Palma TV, da Silva AD, Miron VV, et al. Caffeic acid attenuates neuroinflammation and cognitive impairment in streptozotocin-induced diabetic rats: Pivotal role of the cholinergic and purinergic signaling pathways. The Journal of Nutritional Biochemistry 2023;115.

30. Feng Y, Chu A, Luo Q, Wu M, Shi X, Chen Y. The Protective Effect of Astaxanthin on Cognitive Function via Inhibition of Oxidative Stress and Inflammation in the Brains of Chronic T2DM Rats. Front Pharmacol 2018;9:748.

31. Tian X, Liu Y, Ren G, Yin L, Liang X, Geng T, Dang H, et al. Resveratrol limits diabetes-associated cognitive decline in rats by preventing oxidative stress and inflammation and modulating hippocampal structural synaptic plasticity. Brain Res 2016;1650:1-9.

32. Gocmez SS, Sahin TD, Yazir Y, Duruksu G, Eraldemir FC, Polat S, Utkan T. Resveratrol prevents cognitive deficits by attenuating oxidative damage and inflammation in rat model of streptozotocin diabetes induced vascular dementia. Physiol Behav 2019;201:198-207.

33. Hu Y, Zhang Q, Wang JC, Wang J, Liu Y, Zhu LY, Xu JX. Resveratrol improves diabetes-induced cognitive dysfunction in part through the miR-146a-5p/TXNIP axis. Kaohsiung J Med Sci 2023;39:404-415.

34. Ghaderi S, Gholipour P, Komaki A, Shahidi S, Seif F, Bahrami-Tapehebur M, Salehi I, et al. Underlying mechanisms behind the neuroprotective effect of vanillic acid against diabetes-associated cognitive decline: An in vivo study in a rat model. Phytother Res 2024;38:1262-1277.

35. Farbood Y, Ghaderi S, Rashno M, Khoshnam SE, Khorsandi L, Sarkaki A, Rashno M. Sesamin: A promising protective agent against diabetes-associated cognitive decline in rats. Life Sci 2019;230:169-177.

36. Rahigude A, Bhutada P, Kaulaskar S, Aswar M, Otari K. Participation of antioxidant and cholinergic system in protective effect of naringenin against type-2 diabetes-induced memory dysfunction in rats. Neuroscience 2012;226:62-72.

37. Shang J, Che S, Zhu M. Oleuropein Improves Cognitive Dysfunction and Neuroinflammation in Diabetic Rats through the PI3K/Akt/mTOR Pathway. Appl Bionics Biomech 2022;2022:5892463.

38. Wu XL, Deng MZ, Gao ZJ, Dang YY, Li YC, Li CW. Neferine alleviates memory and cognitive dysfunction in diabetic mice through modulation of the NLRP3 inflammasome pathway and alleviation of endoplasmic-reticulum stress. Int Immunopharmacol 2020;84:106559.

39. Xiao Y, Li K, Bian J, Liu H, Zhai X, El-Omar E, Han L, et al. Urolithin A Attenuates Diabetes-Associated Cognitive Impairment by Ameliorating Intestinal Barrier Dysfunction via N-glycan Biosynthesis Pathway. Mol Nutr Food Res 2022;66:e2100863.

40. Kuhad A, Sethi R, Chopra K. Lycopene attenuates diabetes-associated cognitive decline in rats. Life Sci 2008;83:128-134.

41. Miao C, Chen H, Li Y, Guo Y, Xu F, Chen Q, Zhang Y, et al. Curcumin and its analog alleviate diabetes-induced damages by regulating inflammation and oxidative stress in brain of diabetic rats. Diabetology & Metabolic Syndrome 2021;13.

42. Afzal M, Al-Abbasi FA, Kazmi I, Imam SS, Alshehri S, Ghoneim MM, Almalki WH, et al. Fustin Inhibits Oxidative Free Radicals and Inflammatory Cytokines in Cerebral Cortex and Hippocampus and Protects Cognitive Impairment in Streptozotocin-Induced Diabetic Rats. ACS Chemical Neuroscience 2021;12:4587-4597.

43. Kuhad A, Chopra K. Effect of sesamol on diabetes-associated cognitive decline in rats. Experimental Brain Research 2007;185:411-420.

44. Sun X, Li S, Xu L, Wang H, Ma Z, Fu Q, Qu R, et al. Paeoniflorin ameliorates cognitive dysfunction via regulating SOCS2/IRS-1 pathway in diabetic rats. Physiol Behav 2017;174:162-169.

45. Pei B, Sun J. Pinocembrin alleviates cognition deficits by inhibiting inflammation in diabetic mice. J Neuroimmunol 2018;314:42-49.

46. Hu T, Shi JJ, Fang J, Wang Q, Chen YB, Zhang SJ. Quercetin ameliorates diabetic encephalopathy through SIRT1/ER stress pathway in db/db mice. Aging (Albany NY) 2020;12:7015-7029.

47. Dhaliwal J, Dhaliwal N, Akhtar A, Kuhad A, Chopra K. Tetramethylpyrazine Attenuates Cognitive Impairment Via Suppressing Oxidative Stress, Neuroinflammation, and Apoptosis in Type 2 Diabetic Rats. Neurochem Res 2022;47:2431-2444.

48. Wang J, Wang L, Zhou J, Qin A, Chen Z. The protective effect of formononetin on cognitive impairment in streptozotocin (STZ)-induced diabetic mice. Biomed Pharmacother 2018;106:1250-1257.

49. Liu S, Zheng M, Li Y, He L, Chen T. The protective effect of Geniposide on diabetic cognitive impairment through BTK/TLR4/NF-kappaB pathway. Psychopharmacology (Berl) 2020;237:465-477.

50. Miao Y, He T, Zhu Y, Li W, Wang B, Zhong Y. Activation of Hippocampal CREB by Rolipram Partially Recovers Balance Between TNF-alpha and IL-10 Levels and Improves Cognitive Deficits in Diabetic Rats. Cell Mol Neurobiol 2015;35:1157-1164.

51. Kamdi SP, Badwaik HR, Raval A, Ajazuddin, Nakhate KT. Ameliorative potential of phloridzin in type 2 diabetes-induced memory deficits in rats. European Journal of Pharmacology 2021;913.

52. Xianchu L, Kang L, Beiwan D, Huan P, Ming L. Apocynin ameliorates cognitive deficits in streptozotocin-induced diabetic rats. Bratislava Medical Journal 2021;122:78-84.

53. Yin Z, Yu H, Chen S, Ma C, Ma X, Xu L, Ma Z, et al. Asiaticoside attenuates diabetes-induced cognition deficits by regulating PI3K/Akt/NF-κB pathway. Behavioural Brain Research 2015;292:288-299.

54. Nan X, Sun Q, Xu X, Yang Y, Zhen Y, Zhang Y, Zhou H, et al. Forsythoside B ameliorates diabetic cognitive dysfunction by inhibiting hippocampal neuroinflammation and reducing synaptic dysfunction in ovariectomized mice. Front Aging Neurosci 2022;14:974690.

55. Liu J, Zhang Y, Deng X, Yin F. Geniposide decreases the level of Abeta1-42 in the hippocampus of streptozotocin-induced diabetic rats. Acta Biochim Biophys Sin (Shanghai) 2013;45:787-791.

56. Dong X, Kong L, Huang L, Su Y, Li X, Yang L, Ji P, et al. Ginsenoside Rg1 treatment protects against cognitive dysfunction via inhibiting PLC-CN-NFAT1 signaling in T2DM mice. J Ginseng Res 2023;47:458-468.

57. Swain SK, Chandra Dash U, Sahoo AK. Hydrolea zeylanica improves cognitive impairment in high-fat diet fed-streptozotocin-induced diabetic encephalopathy in rats via regulating oxidative stress, neuroinflammation, and neurotransmission in brain. Heliyon 2022;8:e11301.

58. Chen X, Famurewa AC, Tang J, Olatunde OO, Olatunji OJ. Hyperoside attenuates neuroinflammation, cognitive impairment and oxidative stress via suppressing TNF-alpha/NF-kappaB/caspase-3 signaling in type 2 diabetes rats. Nutr Neurosci 2022;25:1774-1784.

59. Gu JX, Cheng XJ, Luo X, Yang X, Pang YP, Zhang XF, Zhang YY, et al. Luteolin Ameliorates Cognitive Impairments by Suppressing the Expression of Inflammatory Cytokines and Enhancing Synapse-Associated Proteins GAP-43 and SYN Levels in Streptozotocin-Induced Diabetic Rats. Neurochem Res 2018;43:1905-1913.

60. Liu Y, Tian X, Gou L, Sun L, Ling X, Yin X. Luteolin attenuates diabetes-associated cognitive decline in rats. Brain Res Bull 2013;94:23-29.

61. Gomaa AA, Makboul RM, Al-Mokhtar MA, Nicola MA. Polyphenol-rich Boswellia serrata gum prevents cognitive impairment and insulin resistance of diabetic rats through inhibition of GSK3beta activity, oxidative stress and pro-inflammatory cytokines. Biomed Pharmacother 2019;109:281-292.

62. Tientcheu JPD, Ngueguim FT, Gounoue RK, Mbock MA, Ngapout R, Kandeda AK, Dimo T. The extract of Sclerocarya birrea, Nauclea latifolia, and Piper longum mixture ameliorates diabetes-associated cognitive dysfunction. Metab Brain Dis 2023;38:2773-2796.

63. Ertas B, Hazar-Yavuz AN, Topal F, Keles-Kaya R, Karakus Ö, Ozcan GS, Taskin T, et al. Rosa canina L. improves learning and memory-associated cognitive impairment by regulating glucose levels and reducing hippocampal insulin resistance in high-fat diet/streptozotocin-induced diabetic rats. Journal of Ethnopharmacology 2023;313.

64. Moghaddam HK, Baluchnejadmojarad T, Roghani M, Khaksari M, Norouzi P, Ahooie M, Mahboobi F. Berberine ameliorate oxidative stress and astrogliosis in the hippocampus of STZ-induced diabetic rats. Mol Neurobiol 2014;49:820-826.

65. Zhang JH, Zhang JF, Song J, Bai Y, Deng L, Feng CP, Xu XY, et al. Effects of Berberine on Diabetes and Cognitive Impairment in an Animal Model: The Mechanisms of Action. Am J Chin Med 2021;49:1399-1415.

66. Shi Y, Sheng P, Zhao Y, Wang X, Xu X, Sun S. Based on Bioinformatics to Explore the Mechanism of "Tangzhiqing" Decoction Alleviating Type 2 Diabetes-associated Cognitive Dysfunction in Mice by Regulating Hippocampal Neuron Apoptosis and Autophagy. Comb Chem High Throughput Screen 2023.

67. Tian R, Liu X, Jing L, Yang L, Xie N, Hou Y, Tao H, et al. Huang-Lian-Jie-Du decoction attenuates cognitive dysfunction of rats with type 2 diabetes by regulating autophagy and NLRP3 inflammasome activation. J Ethnopharmacol 2022;292:115196.

68. Ahmed A, Zeng G, Azhar M, Lin H, Zhang M, Wang F, Zhang H, et al. Jiawei Shengmai San herbal formula ameliorates diabetic associate cognitive decline by modulating AKT and CREB in rats. Phytother Res 2020;34:3249-3261.

69. Chen J, Zhan L, Lu X, Xiao C, Sun N. The Alteration of ZiBuPiYin Recipe on Proteomic Profiling of Forebrain Postsynaptic Density of db/db Mice with Diabetes-Associated Cognitive Decline. J Alzheimers Dis 2017;56:471-489.

70. Chang XH, Liang LN, Zhan LB, Lu XG, Shi X, Qi X, Feng ZL, et al. The effect of Chinese Jinzhida recipe on the hippocampus in a rat model of diabetes-associated cognitive decline. BMC Complement Altern Med 2013;13:161.

71. Shi J, Yin Q, Zhang L, Wu Y, Yi P, Guo M, Li H, et al. Zi Shen Wan Fang Attenuates Neuroinflammation and Cognitive Function Via Remodeling the Gut Microbiota in Diabetes-Induced Cognitive Impairment Mice. Front Pharmacol 2022;13:898360.

72. Yin Q, Zhang L, Han X, Zhang H, Wang F, Qin X, Zhuang P, et al. Zi Shen Wan Fang regulates kynurenine metabolism to alleviate diabetes-associated cognitive impairment via activating the skeletal muscle PGC1alpha-PPARalpha signaling. Phytomedicine 2022;99:154000.

73. Yan W, Zhang M, Yu Y, Yi X, Guo T, Hu H, Sun Q, et al. Blockade of voltage-gated potassium channels ameliorates diabetes-associated cognitive dysfunction in vivo and in vitro. Exp Neurol 2019;320:112988.

74. Li M, Zhou S, Wang G, Qiao L, Yi S, Li T, Pan X, et al. Calpain Inhibitor Calpeptin Improves Alzheimer's Disease-Like Cognitive Impairments and Pathologies in a Diabetes Mellitus Rat Model. Neurotox Res 2022;40:1248-1260.

75. Zhang J, Liu L, Zhang Y, Yuan Y, Miao Z, Lu K, Zhang X, et al. ChemR23 signaling ameliorates cognitive impairments in diabetic mice via dampening oxidative stress and NLRP3 inflammasome activation. Redox Biology 2022;58.

76. Jinawong K, Apaijai N, Wongsuchai S, Pratchayasakul W, Chattipakorn N, Chattipakorn SC. Necrostatin-1 Mitigates Cognitive Dysfunction in Prediabetic Rats With No Alteration in Insulin Sensitivity. Diabetes 2020;69:1411-1423.

77. Du M, Jiang T, He S, Cheng B, Zhang X, Li L, Yang L, et al. Sigma-1 Receptor as a Protective Factor for Diabetes-Associated Cognitive Dysfunction via Regulating Astrocytic Endoplasmic Reticulum-Mitochondrion Contact and Endoplasmic Reticulum Stress. Cells 2023;12.

78. Liang E, Ma M, Wang L, Liu X, Xu J, Zhang M, Yang R, et al. The BET/BRD inhibitor JQ1 attenuates diabetes-induced cognitive impairment in rats by targeting Nox4-Nrf2 redox imbalance. Biochem Biophys Res Commun 2018;495:204-211.

79. Ren T, Liu J, Ge Y, Zhuo R, Peng L, Liu F, Jin X, et al. Chronic oleoylethanolamide treatment attenuates diabetes-induced mice encephalopathy by triggering peroxisome proliferator-activated receptor alpha in the hippocampus. Neurochem Int 2019;129:104501.

80. Peng D, Xia Q, Guan L, Li HY, Qiao LJ, Chen YB, Cai YF, et al. Carnosine Improves Cognitive Impairment Through Promoting SIRT6 Expression and Inhibiting Endoplasmic Reticulum Stress in a Diabetic Encephalopathy Model. Rejuvenation Res 2022;25:79-88.

81. Ndolo RO, Yu L, Zhao Y, Lu J, Wang G, Zhao X, Ren Y, et al. Carnosine-Based Reversal of Diabetes-Associated Cognitive Decline via Activation of the Akt/mTOR Pathway and Modulation of Autophagy in a Rat Model of Type 2 Diabetes Mellitus. Dement Geriatr Cogn Disord 2023;52:156-168.

82. Wang K, Song F, Xu K, Liu Z, Han S, Li F, Sun Y. Irisin Attenuates Neuroinflammation and Prevents the Memory and Cognitive Deterioration in Streptozotocin-Induced Diabetic Mice. Mediators Inflamm 2019;2019:1567179.

83. Kassab S, Begley P, Church SJ, Rotariu SM, Chevalier-Riffard C, Dowsey AW, Phillips AM, et al. Cognitive dysfunction in diabetic rats is prevented by pyridoxamine treatment. A multidisciplinary investigation. Mol Metab 2019;28:107-119.

84. Lee HJ, Yang SJ. Supplementation with Nicotinamide Riboside Reduces Brain Inflammation and Improves Cognitive Function in Diabetic Mice. Int J Mol Sci 2019;20.

85. Muneeb M, Mansou SM, Saleh S, Mohammed RA. Vitamin D and rosuvastatin alleviate type-II diabetes-induced cognitive dysfunction by modulating neuroinflammation and canonical/noncanonical Wnt/beta-catenin signaling. PLoS One 2022;17:e0277457.

86. Hussein HM, Elyamany MF, Rashed LA, Sallam NA. Vitamin D mitigates diabetes-associated metabolic and cognitive dysfunction by modulating gut microbiota and colonic cannabinoid receptor 1. Eur J Pharm Sci 2022;170:106105.

87. Yan W, Guo T, Liu N, Cui X, Wei X, Sun Y, Hu H, et al. Erythropoietin ameliorates cognitive deficits by improving hippocampal and synaptic damage in streptozotocin-induced diabetic mice. Cell Signal 2023;106:110614.

88. Guo T, Yu Y, Yan W, Zhang M, Yi X, Liu N, Cui X, et al. Erythropoietin ameliorates cognitive dysfunction in mice with type 2 diabetes mellitus via inhibiting iron overload and ferroptosis. Exp Neurol 2023;365:114414.

89. Wang M, Yan W, Liu Y, Hu H, Sun Q, Chen X, Zang W, et al. Erythropoietin ameliorates diabetes-associated cognitive dysfunction in vitro and in vivo. Sci Rep 2017;7:2801.

90. Wu Y, Wu C, Ye L, Wang B, Yuan Y, Liu Y, Zheng P, et al. Exogenous fibroblast growth factor 1 ameliorates diabetes-induced cognitive decline via coordinately regulating PI3K/AKT signaling and PERK signaling. Cell Commun Signal 2020;18:81.

91. Zhao L, Jiang H, Xie J, Shen D, Yi Q, Yan J, Li C, et al. Effects of Fibroblast Growth Factor 21 on Lactate Uptake and Usage in Mice with Diabetes-Associated Cognitive Decline. Mol Neurobiol 2022;59:5656-5672.

92. Ma S, Zhong D, Ma P, Li G, Hua W, Sun Y, Liu N, et al. Exogenous Hydrogen Sulfide Ameliorates Diabetes-Associated Cognitive Decline by Regulating the Mitochondria-Mediated Apoptotic Pathway and IL-23/IL-17 Expression in db/db Mice. Cell Physiol Biochem 2017;41:1838-1850.

93. Zou W, Yuan J, Tang ZJ, Wei HJ, Zhu WW, Zhang P, Gu HF, et al. Hydrogen sulfide ameliorates cognitive dysfunction in streptozotocin-induced diabetic rats: involving suppression in hippocampal endoplasmic reticulum stress. Oncotarget 2017;8:64203-64216.

94. He J, Chen Z, Kang X, Wu L, Jiang JM, Liu SM, Wei HJ, et al. SIRT1 Mediates H(2)S-Ameliorated Diabetes-Associated Cognitive Dysfunction in Rats: Possible Involvement of Inhibiting Hippocampal Endoplasmic Reticulum Stress and Synaptic Dysfunction. Neurochem Res 2021;46:611-623.

95. Cui Y, Yang M, Wang Y, Ren J, Lin P, Cui C, Song J, et al. Melatonin prevents diabetes-associated cognitive dysfunction from microglia-mediated neuroinflammation by activating autophagy via TLR4/Akt/mTOR pathway. FASEB J 2021;35:e21485.

96. Albazal A, Delshad AA, Roghani M. Melatonin reverses cognitive deficits in streptozotocin-induced type 1 diabetes in the rat through attenuation of oxidative stress and inflammation. J Chem Neuroanat 2021;112:101902.

97. Mizobuchi H, Yamamoto K, Yamashita M, Inagawa H, Kohchi C, Soma GI. Oral Administration of Lipopolysaccharide Prevents Cognitive Impairment in Streptozotocin-induced Diabetic Mice in a Blood Glucose-independent Manner. Anticancer Res 2021;41:4053-4059.

98. Gao M, Ji S, Li J, Zhang S. DL-3-n-butylphthalide (NBP) ameliorates cognitive deficits and CaMKII-mediated long-term potentiation impairment in the hippocampus of diabetic db/db mice. Neurol Res 2019;41:1024-1033.

99. Sood A, Fernandes V, Preeti K, Khot M, Khatri DK, Singh SB. Fingolimod Alleviates Cognitive Deficit in Type 2 Diabetes by Promoting Microglial M2 Polarization via the pSTAT3-jmjd3 Axis. Mol Neurobiol 2023;60:901-922.

100. Wang G, Zhang X, Lu X, Liu J, Zhang Z, Wei Z, Wu Z, et al. Fish oil supplementation attenuates cognitive impairment by inhibiting neuroinflammation in STZ-induced diabetic rats. Aging (Albany NY) 2020;12:15281-15289.

101. Zheng M, Zou C, Li M, Huang G, Gao Y, Liu H. Folic Acid Reduces Tau Phosphorylation by Regulating PP2A Methylation in Streptozotocin-Induced Diabetic Mice. Int J Mol Sci 2017;18.

102. Zhang Y-y, Wang L, Guo H, Han T-t, Chang Y-h, Cui X-c. Levetiracetam attenuates diabetes-associated cognitive impairment and microglia polarization by suppressing neuroinflammation. Frontiers in Pharmacology 2023;14.

103. Rajput MS, Sarkar PD. Modulation of neuro-inflammatory condition, acetylcholinesterase and antioxidant levels by genistein attenuates diabetes associated cognitive decline in mice. Chem Biol Interact 2017;268:93-102.

104. Wang K, Song F, Wang H, Wang JH, Sun Y. Quetiapine Attenuates the Neuroinflammation and Executive Function Deficit in Streptozotocin-Induced Diabetic Mice. Mediators Inflamm 2019;2019:1236082.

105. Saha D, Paul S, Gaharwar U, Priya A, Neog A, Singh A, Bk B. Cdk5-Mediated Brain Unfolded Protein Response Upregulation Associated with Cognitive Impairments in Type 2 Diabetes and Ameliorative Action of NAC. ACS Chem Neurosci 2023;14:2761-2774.

106. Piatkowska-Chmiel I, Gawronska-Grzywacz M, Popiolek L, Herbet M, Dudka J. The novel adamantane derivatives as potential mediators of inflammation and neural plasticity in diabetes mice with cognitive impairment. Sci Rep 2022;12:6708.

107. Ma H, Jiang T, Tang W, Ma Z, Pu K, Xu F, Chang H, et al. Transplantation of platelet-derived mitochondria alleviates cognitive impairment and mitochondrial dysfunction in db/db mice. Clin Sci (Lond) 2020;134:2161-2175.
